# Exploring metabolic dynamics during the fermentation of sea buckthorn beverage: comparative analysis of volatile aroma compounds and non-volatile metabolites using GC–MS and UHPLC–MS

**DOI:** 10.3389/fnut.2023.1268633

**Published:** 2023-09-07

**Authors:** Bo Peng, Jingjing Li, Chunhui Shan, Wenchao Cai, Qin Zhang, Xinxin Zhao, Shi Li, Jing Wen, Lin Jiang, Xinquan Yang, Fengxian Tang

**Affiliations:** ^1^School of Food Science, Shihezi University, Shihezi, Xinjiang, China; ^2^Key Laboratory for Processing and Quality Safety Control of Specialty Agricultural Products of Ministry of Agriculture and Rural Affairs, Shihezi University, Shihezi, Xinjiang, China; ^3^Key Laboratory for Food Nutrition and Safety Control of Xinjiang Production and Construction Corps, Shihezi University, Shihezi, Xinjiang, China

**Keywords:** sea buckthorn, yeast, fermentation, untargeted metabolomics, aroma

## Abstract

Sea buckthorn has a high nutritional value, but its sour taste and foul odor make it unpalatable for consumers. In this study, we analyzed the metabolite changes occurring during the yeast-assisted fermentation of sea buckthorn juice using the HeadSpace Solid-Phase Microextraction Gas Chromatography–Mass Spectrometry (HS-SPME-GC–MS) and Ultra-High Performance Liquid Chromatography-Mass Spectrometry (UHPLC–MS) techniques. A total of 86 volatile aroma compounds were identified during the fermentation process. The content of total volatiles in sea buckthorn juice increased by 3469.16 μg/L after 18 h of fermentation, with 22 compounds showing elevated levels. Notably, the total content of esters with fruity, floral, and sweet aromas increased by 1957.09 μg/L. We identified 379 non-volatile metabolites and observed significant increases in the relative abundance of key active ingredients during fermentation: glycerophosphorylcholine (increased by 1.54), glutathione (increased by 1.49), L-glutamic acid (increased by 2.46), and vanillin (increased by 0.19). KEGG pathway analysis revealed that amino acid metabolism and lipid metabolism were the primary metabolic pathways involved during fermentation by *Saccharomyces cerevisiae*. Fermentation has been shown to improve the flavor of sea buckthorn juice and increase the relative content of bioactive compounds. This study provides novel insights into the metabolic dynamics of sea buckthorn juice following yeast fermentation through metabolomics analysis. These findings could serve as a theoretical foundation for further studies on the factors influencing differences in yeast fermentation.

## 1. Introduction

Sea buckthorn (*Hippophae rhamnoides* L.), also known as seaberry or sandthorn, is a deciduous shrub belonging to the family *Elaeagnaceae* (1). It has an exceptional ability to withstand drought, and it can thrive even under poor soil conditions such as high salinity or sandy soil. Hence, sea buckthorn plays a crucial role in soil and water conservation and has valuable windbreak and sand-fixation functions (2).

Sea buckthorn is widely distributed in the Northwestern and Northern regions of China (3). Mature sea buckthorn fruits are typically oval in shape and yellow, orange, or red in color. These fruits are also rich in various nutrients and bioactive compounds and have gained significant attention due to their nutritional and health-promoting properties (1). Sea buckthorn has high levels of flavonoid compounds, which have been found to be effective in the prevention and treatment of chronic diseases such as cancer, diabetes, and cardiovascular diseases (4, 5). However, due to its high water content (approximately 70%), sea buckthorn is susceptible to mechanical damage and microbial spoilage and is thus not suitable for room-temperature storage. Additionally, sea buckthorn fruits have a sour and astringent taste, which negatively affects the flavor of sea buckthorn puree. This significantly hampers the production and development of sea buckthorn-based beverages (6).

Yeasts have several key applications in the food industry because they convert sugars into ethanol and CO_2_, thereby imparting unique flavors to food products (7). Additionally, during the fermentation process, yeasts generate active components that can break down certain macromolecules and enhance their digestibility (8). The presence of various essential nutrients in yeast cells can also enhance the nutritional value of these products; improve their flavor, aroma, and texture; and augment their nutritional characteristics while reducing the levels of anti-nutrients and toxins (9). Wang et al. (10) discovered that the commercial yeast strain RW can significantly enhance the quality, body, and antioxidant properties of Goji-Jujube composite wine. Furthermore, Han et al. (11) found that the commercial yeast strains RV171, Lalvin 71B, and BV818 produced higher levels of total flavonoids and total phenols during the fermentation of green plum wine. Regarding antioxidant ability, RV171-and BV818-fermented green plum wine exhibited the highest ABTS• + and DPPH• radical scavenging rate, respectively.

Metabolomics is a scientific field that focuses on the study of organic acids and other low-molecular-weight metabolites in biological samples in the presence of different growth environments, periods, and external stimuli (12). In metabolomics, high-throughput detection and data processing techniques enable the integration of information and the detection of biomarkers. Metabolomics has found wide applications in food research (13). Duan et al. (14) utilized UHPLC–MS to analyze the variations in Goji berry fermentation in the presence of different lactic acid bacteria strains. They found variations in the content of quercetin and coumarin among berries fermented with different strains, highlighting the strain-specificity of flavonoid metabolism. Zhang et al. (15) employed UHPLC/Q Exactive/MS technology to detect a total of 401 metabolites in chickpea soy milk fermented by lactic acid bacteria for 0 h, 12 h, and 24 h. The majority of differential metabolites were generated during the early stages of fermentation. So far, studies on fermented sea buckthorn products have primarily focused on volatile compounds and their aroma characteristics (16). However, there is limited metabolomics-based research on the metabolite dynamics of sea buckthorn during different stages of yeast fermentation (17).

In this study, we utilized sea buckthorn as the raw material and conducted mild fermentation using commercial yeast to investigate metabolite changes in the fermentation broth. A UHPLC-MC untargeted metabolomics approach was employed to study the metabolic components at different stages of sea buckthorn fermentation. Multivariate statistical analysis was performed to screen for differential metabolites and analyze related metabolic pathways. The aim of this study was to provide a suitable research strategy for the chemical characterization and functional component analysis of yeast-fermented sea buckthorn beverages.

## 2. Materials and methods

### 2.1. Preparation of sea buckthorn juice

Sea buckthorn (*Hippophaë rhamnoides L. subsp. sinensis*) was harvested on September 11, 2022, from the 170th Regiment of the Ninth Division of the Xinjiang Production and Construction Corps in Xinjiang, China. Frozen berry was gathered and sent to juice processing plant promptly. Sea buckthorn berries were first cleaned with distilled water. Then, the berries were added to a blender along with distilled water (weight equal to 1.5 times the weight of the berries) for crushing. The seeds were removed after filtration through a piece of gauze. Then, the sugar content and pH value of the sea buckthorn juice were adjusted to 13°Brix and 4.0, respectively, using sucrose and anhydrous sodium carbonate (18). The sea buckthorn juice was then subjected to pasteurization at 75°C for 20 min, followed by cooling to room temperature (20–30°C).

### 2.2. Preparation of fermented sea buckthorn juice

Commercial yeast powder RV171 (Hubei Angel Yeast Co., Ltd.) was dissolved in a 5% glucose solution at a ratio of 1: 10 (v/v) to obtain a yeast suspension. The yeast suspension was then activated by incubating it in a water bath at 38°C for 25 min. Subsequently, the sea buckthorn juice was inoculated with the yeast suspension (0.03% v/v of the sea buckthorn juice) and fermented at 23°C. Three groups of samples were collected for analysis: high-temperature short-time sterilization sterilized sea buckthorn fermentation liquid without yeast inoculation (FSJ0), samples fermented for 9 h after yeast inoculation (FSJ9), and samples fermented for 18 h after yeast inoculation (FSJ18). A quality control sample was also prepared by combining portions from each sample. Metabolomic analysis was performed using UHPLC–MS.

### 2.3. HS-SPME–GC–MS analysis and calculation of odor activity values

The method for extracting volatile compounds from sea buckthorn berries was modified from (19). First, 5 mL sea buckthorn juice was mixed with 1.5 g NaCl and 100 μL of (S)-(+)-2-Octanol as an internal standard in a 20 mL headspace vial. A manual SPME sampler with a 50/30 μm DVB/CAR/PDMS fiber was used for extraction for 30 min. The sampler was inserted into the GC injector and thermally desorbed for 5 min at 240°C. Analysis was performed using GC–MS (Agilent 7,890-5977A) as described by Cai et al. (19), with slight modifications. An HP-INNOWAX (30 m × 0.25 mm × 0.25 μm) column was used, with helium as the carrier gas (1 mL/min). The temperature program was as follows: 40°C for 5 min, 3°C/min to 151°C for 3 min, and 10°C/min to 250°C for 5 min. An electron ionization source was used; its parameters were as follows: temperature of 230°C, electron energy of 70 eV, and mass scan range of 35 to 750 amu. Volatile compounds were identified using the NIST 2017 database based on retention indices and mass spectra. The content of each compound was determined using (S)-(+)-2-Octanol as the internal standard. The volatile compounds were quantified without considering response factors and recovery rates, as shown below:


$$C_{x}=\frac{A_{x}}{A_{s}\times m_{x}}\times m_{s}$$

Here, Cx represents the concentration of the target compound X (μg/kg), Ax represents the peak area of compound X, As represents the peak area of the internal standard s, mx represents the sample mass (kg), and ms represents the mass of the internal standard s (μg).

The odor activity value (OAV) of volatile compounds was calculated based on the ratio of the content of the volatile flavor substance to its threshold value using the following mathematical equation:


$$\mathrm{OAV}=\frac{C_{x}}{{\mathrm{OT}}_{x}}$$

Here, Cx represents the concentration of the target compound X (μg/kg) and OTx represents the threshold value for compound X.

### 2.4. UHPLC/MS analysis

Metabolite analysis was performed using UHPLC/MS (Thermo Scientific, Thermo Fisher Scientific Inc., Waltham, Massachusetts, United States) as described previously, with some modifications (20). Sample preparation: 200 μL of the sample was weighed and mixed with 200 μL of ultrapure water and 800 μL of methanol-acetonitrile (1, 1, v/v). The mixture was vortexed and subjected to ultrasonic extraction (5°C, 40 KHz, 30 min), followed by centrifugation (13,000 r/min, 4°C, 15 min). The supernatant was then filtered (0.22-μm filter) and used for subsequent analysis. The chromatographic conditions were as follows. An ACQUITY UPLC HSS T3 column (100 mm × 2.1 mm, 1.8 μm, Waters, Milford, United States) was used. Mobile phase A consisted of 95% water and 5% acetonitrile (containing 0.1% formic acid), while mobile phase B consisted of 47.5% acetonitrile and 5% water (containing 0.1% formic acid). The injection volume was 2 μL, and the column temperature was set to 40°C. The sequence of flow-phase gradient elution was 75.5% A and 24.5% B for 3.5 min at a flow rate of 0.4 mL/min; 35% A and 65% B for 5 min at a flow rate of 0.4 mL/min; 100% B for 5.5 min at a flow rate of 0.4 mL/min; 100% B for 7.4 min at a flow rate of 0.6 mL/min; 48.5% A and 51.5% B for 7.6 min at a flow rate of 0.6 mL/min; 100% A for 7.8 min at a flow rate of 0.5 mL/min; 100%; and 100% A for 19 min at a flow rate of 0.4 mL/min. For MS, the sample was subjected to electrospray/ionization, and mass spectrometric signals were collected in the negative ion scanning mode. The scanning range was 70 to 1,050 m/z. The sheath gas flow rate was 50 arb, and the auxiliary gas flow rate was 10 arb. The heating temperature was set to 425°C, while the capillary temperature was maintained at 325°C. The positive mode spray voltage was 3,500 V, the negative mode spray voltage was –3,500 V, and the S-Lens voltage was set to 50 V. Collision energies of 20, 40, and 60 were applied. The resolution for full MS was set to 60,000, while that for MS2 was set to 7,500.

Metabolite identification was carried out using Progenesis QI software (Version 3.0) (MS) and MS/MS data were matched against a metabolite database, with a mass accuracy error set to be <10 ppm. Simultaneously, metabolite identification was based on the matching scores of the secondary mass spectrometry data.

### 2.5. Statistical analysis

Principal component analysis (PCA) was employed for data quality control (QC) and quality assurance (QA) analysis. PCA and partial least squares-discriminant analysis (PLS-DA) were performed based on R2 and Q2 values, respectively. In the PLS-DA model, differential metabolites were identified based on principal components with Variable Importance in Projection (VIP) values >2. These differential metabolites were analyzed using the KEGG database to identify related metabolic pathways. Analysis of variance (ANOVA) was conducted using SPSS software. Heatmaps and bar graphs were generated using Origin 9.8.5 and TBTOOLS. All samples were measured with three biological replicates.

## 3. Results and discussion

### 3.1. Changes in volatiles during the production of yeast-fermented sea buckthorn beverages

#### 3.1.1. Content of volatile aroma compounds detected during fermentation

**Figure 1:**
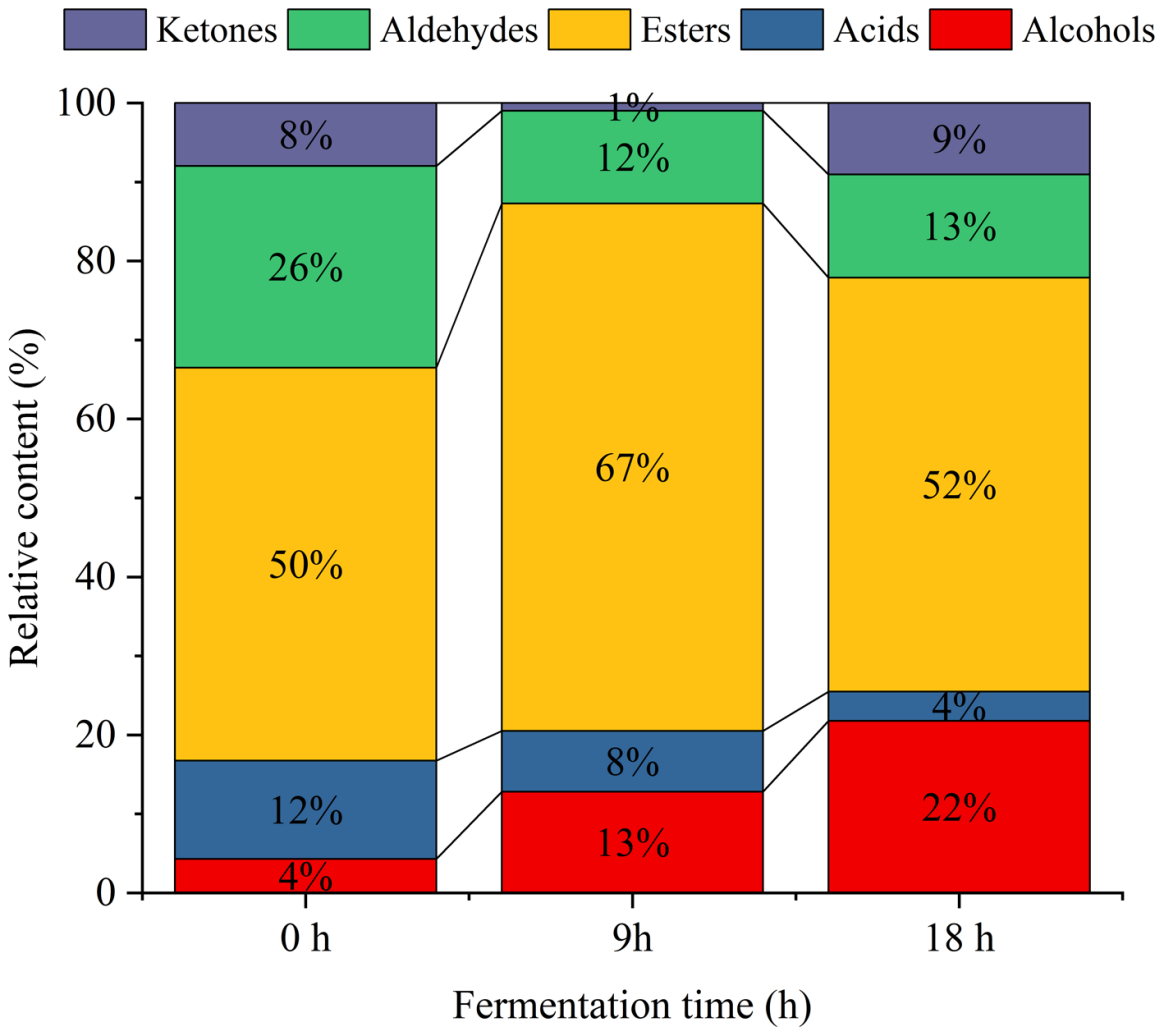
Stacking histogram of volatile substances in sea buckthorn juice fermented by yeast.

The composition and concentration of volatile aroma compounds are key determinants of the flavor and quality of fermented products, directly influencing their sensory characteristics (21, 22). The volatile components produced by yeasts during the fermentation of sea buckthorn juice exhibit significant variations and shape its flavor profile. Therefore, fermentation-related changes in the types and contents of volatile flavor compounds in yeast-fermented sea buckthorn juice were examined using the HS-SPME-GC–MS technique. As shown in Table 1, a total of 86 volatile aroma compounds were detected in the yeast-fermented sea buckthorn beverage. These compounds included 45 esters, 16 alcohols, 12 aldehydes, 8 acids, and 5 ketones. Notably, 51, 61, and 73 volatile aroma compounds were detected in sea buckthorn juice at 0 h, 9 h, and 18 h of fermentation, respectively. The content of volatile aroma compounds also increased with the duration of fermentation, demonstrating a significant (*p* < 0.05) increase in the total amount of volatile aroma compounds in sea buckthorn juice following yeast fermentation. The percentage stacked bar chart in Figure 1 depicted the changes in various volatile compounds during the yeast fermentation of sea buckthorn beverages. The relative concentrations of alcohols, esters, ketones, and phenolic compounds in sea buckthorn juice increased during yeast fermentation, and this increase was especially significant for the esters and alcohols. Conversely, the relative concentrations of acids and aldehydes decreased. These findings align with previous studies from the literature (23).

**Table 1:** Changes in volatile aroma content during fermentation of sea buckthorn juice.

Peak code	CAS	Compounds	Content (μg/L)		
			0 h	9 h	18 h
**Alcohols**					
A1	123-51-3	3-Methyl-1-butanol	4.52 ± 0.09^c^	5.71 ± 0.09^b^	6.57 ± 0.16^a^
A2	19,549-98-5	3,6-Dimethyl-1-heptyn-3-ol	-	-	139.11 ± 3.49^a^
A3	600-36-2	2,4-Dimethyl-3-pentanol	-	-	281.02 ± 7.05^a^
A4	111-27-3	1-Hexanol	47.75 ± 0.95^c^	52.74 ± 0.81^b^	64.7 ± 1.62^a^
A5	111-70-6	1-Heptanol	-	-	5.66 ± 0.14^a^
A6	4,630-06-2	6-Methyl-5-hepten-2-ol	-	45.83 ± 0.7^a^	-
A7	58,175-57-8	2-Propyl-1-pentanol	-	-	130.79 ± 3.28^a^
A8	104-76-7	2-Ethylhexanol	-	117.85 ± 1.81^b^	193.47 ± 5.8^a^
A9	6,191-71-5	cis-4-Hepten-1-ol	-	-	193.22 ± 4.85^a^
A10	5,271-38-5	2-(Methylthio)ethanol	-	-	11.44 ± 0.29^a^
A11	111-87-5	1-Octanol	18.29 ± 0.37^c^	23.84 ± 0.37^a^	19.33 ± 0.48^b^
A12	143-08-8	1-Nonanol	-	-	22.66 ± 0.57^a^
A13	6,126-49-4	tetrahydro-5-methylfuran-2-methanol	-	444.78 ± 6.82^b^	534.18 ± 8.19^a^
A14	2,319-57-5	L-Threitol	-	-	135.93 ± 3.41^a^
A15	100–51-6	Benzyl alcohol	76.84 ± 1.54^b^	71 ± 1.09^c^	80.12 ± 2.01^a^
A16	60-12-8	Phenethyl alcohol	77.09 ± 1.54^a^	65.46 ± 1^b^	77.4 ± 1.94^a^
**Acids**					
B1	79-31-2	Isobutyric acid	77.94 ± 1.56^a^	44.2 ± 0.68^b^	1.41 ± 0.04^c^
B2	15,469-77-9	3-Decenoic acid	-	36.14 ± 0.55^a^	-
B3	503-74-2	Isovaleric acid	17.02 ± 0.34^a^	10.12 ± 0.16^b^	0.76 ± 0.02^c^
B4	1,577-19-1	3-Octenoic acid	401.24 ± 8.02^a^	221.49 ± 3.39^b^	231.21 ± 4.78^b^
B5	19,889-37-3	2-Methyl-2-ethylbutyric acid	130.58 ± 2.61^a^	114.41 ± 1.75^b^	86.63 ± 2.17^c^
B6	142-62-1	Hexanoic acid	26.27 ± 0.53^a^	14.45 ± 0.22^b^	0.69 ± 0.02^c^
B7	111-14-8	Heptanoic acid	-	24.1 ± 0.37^a^	0.33 ± 0.01^b^
B8	124-07-2	Octanoic acid	-	30.82 ± 0.47^a^	2.84 ± 0.07^b^
**Esters**					
C1	105-54-4	Ethyl butyrate	57.96 ± 1.16^c^	70.12 ± 1.07^b^	86.25 ± 2.16^a^
C2	7,452-79-1	Ethyl 2-methylbutyrate	-	47.98 ± 0.74^b^	61.42 ± 1.54^a^
C3	108-64-5	Ethyl isovalerate	1.62 ± 0.03^b^	1.45 ± 0.02^c^	2.38 ± 0.06^a^
C4	56,554-43-9	Methyl 3,6-octadecadiynoate	-	-	53.46 ± 1.34^a^
C5	628-63-7	Amyl acetate	54.6 ± 1.09^a^	-	23.06 ± 0.58^b^
C6	123-92-2	Isoamyl acetate	-	27.84 ± 0.43^b^	33.9 ± 0.52^a^
C7	557-00-6	Propyl isovalerate	83.12 ± 1.66^a^	61.62 ± 0.94^c^	69.57 ± 1.74^b^
C8	2050-09-1	Isoamyl valerate	-	356.98 ± 5.47^a^	-
C9	638-10-8	Ethyl 3,3-dimethylacrylate	38.19 ± 0.76^a^	34.03 ± 0.52^b^	32.35 ± 0.81^c^
C10	16,509-44-7	2-Butenoic acid, 2-Methyl-, ethyl ester, (2Z)-	-	82.87 ± 1.27^a^	-
C11	123-66-0	Ethyl Hexanoate	1.48 ± 0.03^b^	1.26 ± 0.02^c^	2.29 ± 0.06^a^
C12	51,115-64-1	2-Methylbutyl butyrate	31.43 ± 0.63^c^	56.09 ± 0.86^b^	76.84 ± 1.93^a^
C13	2,445-69-4	2-Methylbutyl isobutyrate	-	48.75 ± 0.75^a^	-
C14	27,625-35-0	3-Methylbutyl 2-methyl butanoate	38.41 ± 0.77^c^	50.47 ± 0.77^a^	44.43 ± 1.11^b^
C15	2,445-78-5	2-Methylbutyl 2-methylbutyrate	-	56.11 ± 0.86^a^	33.52 ± 0.84^b^
C16	659-70-1	3-Methylbutyl 3-methylbutanoate	0.52 ± 0.01^b^	0.37 ± 0.01^c^	0.86 ± 0.02^a^
C17	626-77-7	Propyl Hexanoate	182.86 ± 3.66^b^	173.59 ± 2.66^c^	230.26 ± 5.78^a^
C18	540-07-8	Amyl capronate	-	117.99 ± 1.81^a^	-
C19	2,311-46-8	Isopropyl Hexanoate	-	-	453.64 ± 11.38^a^
C20	106-30-9	Ethyl heptanoate	211.6 ± 4.23^c^	221.4 ± 3.39^b^	223.41 ± 5.6^a^
C21	25,415-62-7	pentyl isovalerate	-	283.88 ± 4.35^a^	208.68 ± 5.23^b^
C22	2,173-56-0	Pentyl valerate	-	301.75 ± 4.62^b^	315.72 ± 4.76^a^
C23	105-79-3	Isobutyl hexanoate	142.15 ± 2.84^c^	205.67 ± 3.15^a^	154.93 ± 3.89^b^
C24	626-82-4	Butyl hexanoate	-	-	92.62 ± 2.32^a^
C25	112-06-1	Heptyl acetate	-	358.68 ± 5.5^a^	309.62 ± 7.77^b^
C26	18,267-36-2	Ethyl 3-hydroxy-3-methylbutyrate	6.9 ± 0.14^c^	7.24 ± 0.11^b^	9.24 ± 0.23^a^
C27	2,441-06-7	2-Hydroxy-3-methylbutanoic acid ethyl ester	10.88 ± 0.22^b^	11.75 ± 0.18^b^	14.76 ± 0.37^a^
C28	106-32-1	Ethyl caprylate	11.03 ± 0.22^b^	5.32 ± 0.08^c^	15 ± 0.38^a^
C29	10,032-13-0	Hexyl isovalerate	89.29 ± 1.79^c^	101.33 ± 1.55^a^	94.44 ± 2.37^b^
C30	2,198-61-0	Isopentyl hexanoate	1.8 ± 0.04^c^	1.77 ± 0.03^b^	2.36 ± 0.06^a^
C31	34,495-71-1	Ethyl (Z)-oct-4-enoate	56.42 ± 1.13^a^	52.99 ± 0.81^b^	43.26 ± 1.09^c^
C32	93-58-3	Methyl benzoate	440.48 ± 8.81^a^	337.03 ± 5.17^b^	338.48 ± 7^b^
C33	614-99-3	Ethyl 2-furoate	162.26 ± 3.25^a^	139.18 ± 2.13^b^	120.42 ± 3.02^c^
C34	110-38-3	Ethyl caprate	-	25.62 ± 0.39^b^	26.56 ± 0.67^a^
C35	93-89-0	Ethyl benzoate	6.92 ± 0.14^a^	5.04 ± 0.08^c^	5.94 ± 0.15^b^
C36	7,367-84-2	Ethyl (Z)-4-decenoate	86.89 ± 1.74^a^	-	-
C37	556-24-1	Methyl isovalerate	195.07 ± 3.9^a^	165.79 ± 2.54^b^	119.1 ± 2.99^c^
C38	32,579-81-0	Methyl 3-ethylhexanoate	-	52.7 ± 0.81^a^	42.62 ± 1.07^c^
C39	13,678-60-9	Furfuryl 3-methylbutanoate	94.84 ± 1.9^a^	58.13 ± 0.89^b^	55.69 ± 1.4^c^
C40	101-97-3	Ethyl phenylacetate	240.96 ± 4.82^a^	177.41 ± 2.72^b^	151.73 ± 3.81^c^
C41	25,415-67-2	Ethyl 4-Methylvalerate	-	-	442.63 ± 11.1^a^
C42	615-12-3	2-Furanecarboxylicacidisoamylester	-	328.9 ± 5.04^a^	206.92 ± 5.19^b^
C43	103-38-8	Benzyl isovalerate	34.8 ± 0.7^a^	27.47 ± 0.42^b^	23.76 ± 0.6^c^
C44	94-46-2	Isoamyl benzoate	2.25 ± 0.04^a^	1.86 ± 0.03^b^	1.73 ± 0.04^c^
C45	140-26-1	Phenylethyl isovalerate	319.96 ± 6.4^b^	253 ± 3.88^c^	337.93 ± 8.48^a^
**Aldehydes**					
D1	590-86-3	Isovaleraldehyde	-	-	40.67 ± 1.02^a^
D2	66-25-1	Hexanal	16.55 ± 0.33^c^	249.87 ± 3.83^a^	205.01 ± 3.14^b^
D3	111-71-7	Heptaldehyde	104.38 ± 2.09^a^	-	-
D4	124-13-0	Octanal	86.71 ± 1.73^c^	95.83 ± 1.47^b^	155.89 ± 3.91^a^
D5	18,829-55-5	Trans-2-Heptenal	69.42 ± 1.39^a^	-	-
D6	124-19-6	1-Nonanal	41.08 ± 0.82^b^	42.56 ± 0.65^b^	85.36 ± 2.14^a^
D7	112-31-2	Decyl aldehyde	433.71 ± 8.67^a^	328.92 ± 5.04^c^	415 ± 10.41^b^
D8	98-01-1	Furfural	37.96 ± 0.76^a^	-	-
D9	43-03-5	Trans,trans-2,4-heptadienal	393.39 ± 7.87^a^	-	-
D10	100-52-7	Benzaldehyde	19.31 ± 0.39^c^	40.71 ± 0.62^b^	234.55 ± 5.88^a^
D11	122-78-1	Phenylacetaldehyde	-	-	2.14 ± 0.05^a^
D12	15,764-16-6	2,4-Dimethylbenzaldehyde	135.58 ± 2.71^a^	-	-
**Ketones**					
E1	431-03-8	2,3-Butanedione	-	-	407.45 ± 10.22^a^
E2	111-13-7	2-Octanone	255.64 ± 5.11^a^	-	261.51 ± 7.85^a^
E3	513-86-0	Acetoin	-	-	46.44 ± 1.16^a^
E4	110-93-0	6-Methyl-5-hepten-2-one	70.33 ± 1.41^a^	63.94 ± 0.98^b^	72.04 ± 1.81^a^
E5	78-59-1	Isophorone	91.86 ± 1.84^a^	-	-

“-” represents not detected or below instrument detection limit.

Esters comprised 50% of the total volatile compounds in unfermented sea buckthorn juice. After 9 h of fermentation, the percentage of esters significantly increased (*p* < 0.05) to 67%. At 18 h of fermentation, the relative content of esters decreased to 52% of the total volatile compounds. However, the total content of esters remained significantly higher than that in unfermented sea buckthorn juice. This could be attributed to the synthesis of esters by yeasts through pathways involving lipases, alcohol acyltransferases (24). At 18 h of fermentation, the contents of ethyl 2-methylbutyrate, isoamyl acetate, isoamyl butyrate, 2-methylbutyrate-2-methylbutyl, propyl caproate, isopropyl caproate, heptyl acetate, ethyl caproate, ethyl isohexanoate, and isoamyl furoate increased significantly (*p* < 0.05). Conversely, the contents of methyl isovalerate, propyl isovalerate, ethyl cis-4-octenoate, pentyl acetate, methyl benzoate, 2-furoic acid ethyl ester, (Z)-4-decenoic acid ethyl ester, furfuryl isovalerate, ethyl phenylacetate, and isoamyl benzoate were significantly reduced (*p* < 0.05).

Yeasts metabolize alcohol compounds during the fermentation process, generating them through two pathways: catabolic and anabolic (25, 26). Glucose metabolism produces keto acids, which are then decarboxylated and dehydrogenated into higher alcohols by acetolactate decarboxylase and dehydrogenase, respectively. On the other hand, catabolic metabolism involves amino acids converting into keto acids through transamination, which are then transformed into aldehydes under the action of transaminases and decarboxylases, ultimately resulting in higher alcohols (26). Consequently, the proportion of alcohol compounds steadily increases during the fermentation of sea buckthorn beverage by yeasts. Consequently, the content of alcohols increased from 224.48 μg/L to 1895.60 μg/L during the yeast fermentation of sea buckthorn juice. This increase in alcohol content contributed to the enriched aroma of the yeast-fermented sea buckthorn beverage. Notably, phenethyl alcohol, benzyl alcohol, and 2-ethylhexanol provided fruity, floral, and sweet aromas, respectively (27). The acid content in the beverage decreased from 12 to 4% during fermentation, with a reduction of 329.18 μg/L at 18 h versus the pre-fermentation period. The compounds showing the most significant reductions were isobutyric acid, isovaleric acid, octenoic acid, 2-ethyl-2-methylbutyric acid, and capric acid. Additionally, yeasts produced carbonyl compounds such as aldehydes and ketones during fermentation (28). Notably, significantly increased levels (*p* < 0.05) of 6-methyl-5-hepten-2-one, 2,3-butanedione, 2-Octanone, and 3-hydroxy-2-butanone were observed during fermentation, contributing to the creamy aroma and fruity flavor of the yeast-fermented sea buckthorn beverage (27).

#### 3.1.2. Characteristic aroma compounds detected during fermentation

During the yeast fermentation of sea buckthorn beverages, a total of 30 volatile aroma compounds with OAV > 1 were detected (Table 2). These compounds included 15 esters, 6 aldehydes, 4 ketones, 4 acids, and 3 alcohols. Esters were the predominant flavor compounds in yeast-fermented sea buckthorn beverages, consistent with findings from Wang et al. (6).

**Table 2:** OAV >1 for the volatile compounds in sea buckthorn fermented by yeast.

Compounds	Fragrance properties^1^	OT^2^	Fermentation time		
		(μg/kg)	0 h	6 h	18 h
**Alcohols**					
3-Methyl-1-butanol	Apple brandy aroma and spicy flavour	4	1.13	1.43	1.64
1-Hexanol	Slightly winey, fruity and fatty notes	5.6	8.53	9.45	11.52
1-Heptanol	Oily and spicy aroma, almost citrusy.	5.4	-	-	1.04
Acids					
Isobutyric acid	Acidic sour cheese dairy buttery rancid	10	7.79	4.42	0.14
Isovaleric acid	Sour stinky feet sweaty cheese tropical	12	1.42	0.84	0.063
Hexanoic acid	Sour fatty sweat cheese	0.06	26.27	14.45	0.69
Heptanoic acid	Rancid sour cheesy sweat	22	-	1.1	0.015
**Esters**					
Ethyl butyrate	Like apple or pineapple.	0.9	64.4	78.18	95.51
2-methylbutyl butyrate	Like banana or pears	15	2.1	3.75	5.11
2-methyl-, 3-methylbutyl ester	Sweet fruity citrus cherry blueberry apple	8.6	4.47	5.89	5.15
Ethyl 2-methylbutyrate	Sharp sweet green apple fruity	0.01	-	3703.27	4709.14
Ethyl heptanoate	Fruity pineapple cognac rum wine	1.9	111.37	116.92	117.19
1-Pentyl n-valerate	Ripe fruity apple	26	-	11.64	11.98
Ethyl isovalerate	Fruity sweet apple pineapple tutti frutti	0.01	161.53	145.11	237.7
Methyl benzoate	Phenolic wintergreen almond floral cananga	73	6.03	4.63	4.61
Ethyl caprate	Sweet waxy fruity apple grape oily brandy	5	-	5.14	5.29
Methyl isovalerate	Strong apple fruity pineapple	4.4	44.33	37.8	26.98
Ethyl phenylacetate	Sweet floral honey rose balsam cocoa	155.55	1.55	1.14	0.97
Ethyl 4-methylval ester	Fruity	0.003	-	-	147.05
Isoamyl acetate	Banana-like odor	0.15	-	186.25	226.73
Propyl isovalerate	Bitter sweet apple fruity	8.7	9.55	7.11	7.97
Ethyl 3,3-dimethylacrylate	Fruity, sweet orange	25	1.53	1.37	1.29
**Aldehydes**					
Isovaleraldehyde	Ethereal aldehydic chocolate peach fatty	1.1	-	-	36.85
Heptaldehyde	Fresh aldehydic fatty green herbal wine-Lee ozone	2.8	37.28	-	-
Octanal	Fatty-orange odor	0.58	147.71	163.8	264.68
trans-2-Heptenal	Pungent green vegetable fresh fatty	40	1.74	-	-
1-Nonanal	Waxy aldehydic rose fresh orris orange peel fatty peely	1.1	37.35	38.82	77.35
Decyl aldehyde	Flower, wax	3	144.57	110.01	137.87
**Ketones**					
2,3-Butanedione	Strong butter sweet creamy pungent caramel	0.06	-	-	6883.01
2-Octanone	Earthy weedy natural woody herbal	50.2	5.09	-	5.21
Acetoin	Buttery	14	-	-	3.31
6-Methyl-5-hepten-2-one	Citrus green musty lemongrass apple	68	1.03	0.94	1.06

“-” indicates an OAV value < 1. ^1^Fragrance properties reference flavornet and human odor space, retrieved from http://www.flavornet.org/index.html. ^2^Odour thresholds reference to a book: L.J. van Gemert, Zeist (2011). Odour Thresholds Compilations of odour threshold values in air, water and other media.

Esters, the primary group of aroma compounds in yeast-fermented sea buckthorn beverages, typically exhibit fruity and floral aromas. They have high OAVs and contribute greatly to the overall flavor of the fermented beverage. Unfermented sea buckthorn juice contained a total of 22 aroma compounds with an OAV > 1. Among them, 10 were esters, including ethyl butyrate (OAV = 64.40), ethyl isovalerate (OAV = 161.53), isoamyl butyrate (OAV = 2.10), and ethyl heptanoate (OAV = 111.37), which imparted fruity flavors of apple, pineapple, and banana to the sea buckthorn juice. Additionally, methyl benzoate (OAV = 6.03) and ethyl phenylacetate (OAV = 1.55) contributed to the strong floral and honey aroma of the juice. During yeast fermentation, the ester content increased, but the content of methyl benzoate and ethyl phenylacetate significantly decreased (*p* < 0.05). At 9 h of fermentation, 15 esters with an OAV > 1 were detected. Notably, ethyl 2-methylbutyrate (OAV = 3703.27), isoamyl acetate (OAV = 186.25), isoamyl butyrate (OAV = 3.75), and ethyl caprate (OAV = 5.14) exhibited significantly increased OAVs (*p* < 0.05), intensifying the fruity and coconut aroma of the beverage. The ester content of the yeast-fermented sea buckthorn beverage peaked (4561.79 μg/L) at 18 h of fermentation, leading to a significant enhancement of its fruity and coconut aroma.

Alcohols were the second most important group of aroma compounds in the yeast-fermented sea buckthorn beverage. The content of alcohols significantly increased (*p* < 0.05) during fermentation, going from 224.48 μg/L to 1895.60 μg/L. A total of 10 alcohols were detected during the fermentation of the beverage, with isoamyl alcohol, n-hexanol, and n-heptanol having OAVs greater than 1. The content of these alcohols increased gradually during fermentation and peaked at 18 h of fermentation, reaching 6.57 μg/L, 64.70 μg/L, and 5.66 μg/L, respectively. These alcohols added fruity, mellow, and fatty aromas to the yeast-fermented sea buckthorn beverage (29, 30). Additionally, at 18 h of fermentation, the content of 1-nonanol, 2-methylthioethanol, and 2,4-dimethyl-3-pentanol increased (*p* < 0.05) by 22.66 μg/L, 11.44 μg/L, and 281.02 μg/L, respectively, compared to 0 h of fermentation. These alcohols contributed rose wax and fruity fatty aromas to the yeast-fermented sea buckthorn beverage (31).

Sea buckthorn juice contained a total of 8acids, including isobutyric, isovaleric, and hexanoic acids, which contributed to a foul odor and sweat-like smell, negatively impacting the flavor of the juice (32). However, after yeast fermentation, the content of these acids decreased significantly (*p* < 0.05). At 18 h of fermentation, their respective contents had decreased by 76.53 μg/L, 16.26 μg/L, and 25.58 μg/L. Additionally, their OAVs fell below the threshold, indicating that yeast fermentation successfully mitigated these unpleasant odors and improved the quality of sea buckthorn juice. The propionic, valeric, heptanoic and nonanoic acids in durian fruit, which also have an unpleasant odor, are metabolized to trace levels during yeast fermentation and produce the corresponding esters such as ethyl propionate and ethyl hexanoate (33).

Yeast fermentation resulted in the production of several aldehydes and ketones. Aldehydes such as isovaleraldehyde, n-octanal, and nonanal with OAVs greater than 1 exhibited a significant increase (*p* < 0.05) after 18 h of fermentation. Specifically, their respective contents increased by 40.67 μg/L, 69.18 μg/L, and 44.28 μg/L relative to the initial stage of fermentation. This led to the infusion of apple and peach aromas, as well as fatty wax and citrus flavors, into the sea buckthorn juice (31). Furthermore, 2,3-butanedione, 2-Octanone, 3-hydroxy-2-butanone, 6-methyl-5-hepten-2-one, and other ketones with OAVs greater than 1 contributed fruity and creamy aromas to the yeast-fermented sea buckthorn beverage. Their content gradually increased during the fermentation process and reached peak levels at 18 h, i.e., 407.45 μg/L, 261.51 μg/L, 46.44 μg/L, and 72.04 μg/L, respectively.

### 3.2. Changes in non-volatile metabolites during the production of yeast-fermented sea buckthorn beverages

#### 3.2.1. Metabolite analysis of yeast-fermented sea buckthorn beverages

**Figure 2:**
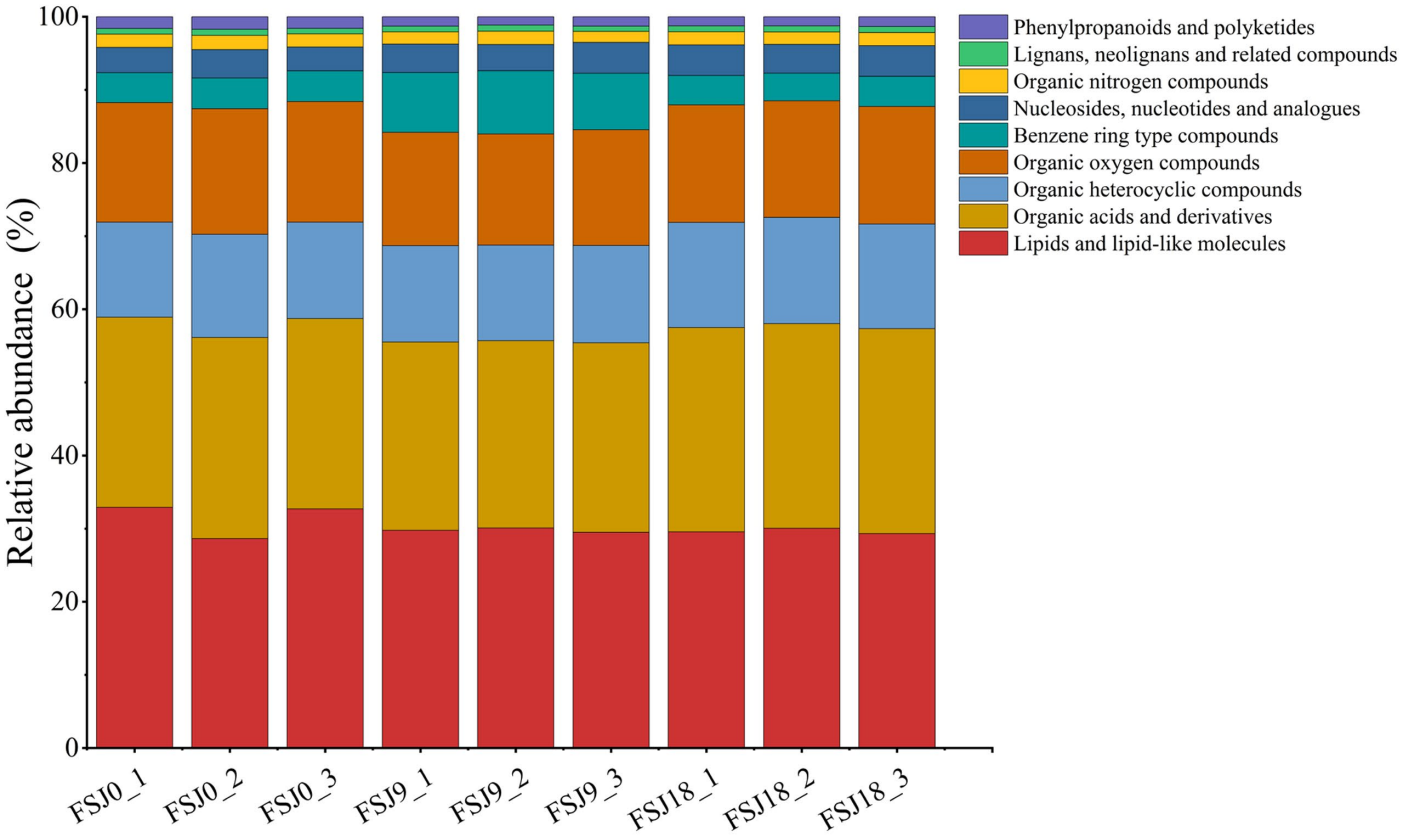
Histogram showing the percentage accumulation of changes in non-volatile metabolites in sea buckthorn juice fermented by yeast based on HMDB super classes.

Metabolomics involves the high-throughput detection, integration, and identification of small-molecule metabolites under different growth conditions and enables the identification of biomarkers. It is currently widely applied in the food industry (34). The metabolic changes in sea buckthorn juice during yeast fermentation were monitored using UPLC/Q Exactive/MS. A total of 379 metabolite ion signals (156 metabolites in the positive ion mode and 123 in the negative ion mode) were detected across the three samples (FSJ0, FSJ9, and FSJ18; Supplementary Table S1). All metabolites detected at 0 h, 9 h, and 18 h of fermentation were matched against the human metabolome database (HMDB). These metabolites belonged to 9 primary classifications, 28 secondary classifications, and 47 tertiary classifications, with some metabolites remaining unconfirmed. As shown in Figure 2, at the first level of classification, the non-volatile metabolite categories with an average relative abundance >5% were lipids and lipophilic molecules (29.22%), organic acids and derivatives (28.68%), organic heterocyclic compounds (16.16%), and organic oxygen-containing compounds (13.77%). The non-volatile metabolite categories with an average relative abundance <5% were aromatic compounds (3.94%), nucleosides, nucleotides, and analogs (3.94%), organic nitrogen-containing compounds (2.44%), phenolic and polyketone compounds (1.30%), and lignans, neolignans, and related compounds (0.85%). Notably, phenolic and polyketone compounds exhibited decreasing levels during fermentation, with the content reducing from 77.18 to 75.24 (*p* < 0.05). Aromatic compounds showed the highest content in the 9-h fermented samples, with their levels reaching 117.84. Furthermore, the non-volatile metabolite content in fermented sea buckthorn beverage at 18 h was 1826.29 ± 6.70, significantly higher (*p* < 0.05) than that at 0 h and 9 h of fermentation. These results indicate that compounds undergo mutual conversion during fermentation, and sea buckthorn juice produces more non-volatile metabolites after yeast fermentation.

#### 3.2.2. PCA and PLS-DA of non-volatile metabolites during fermentation

**Figure 3:**
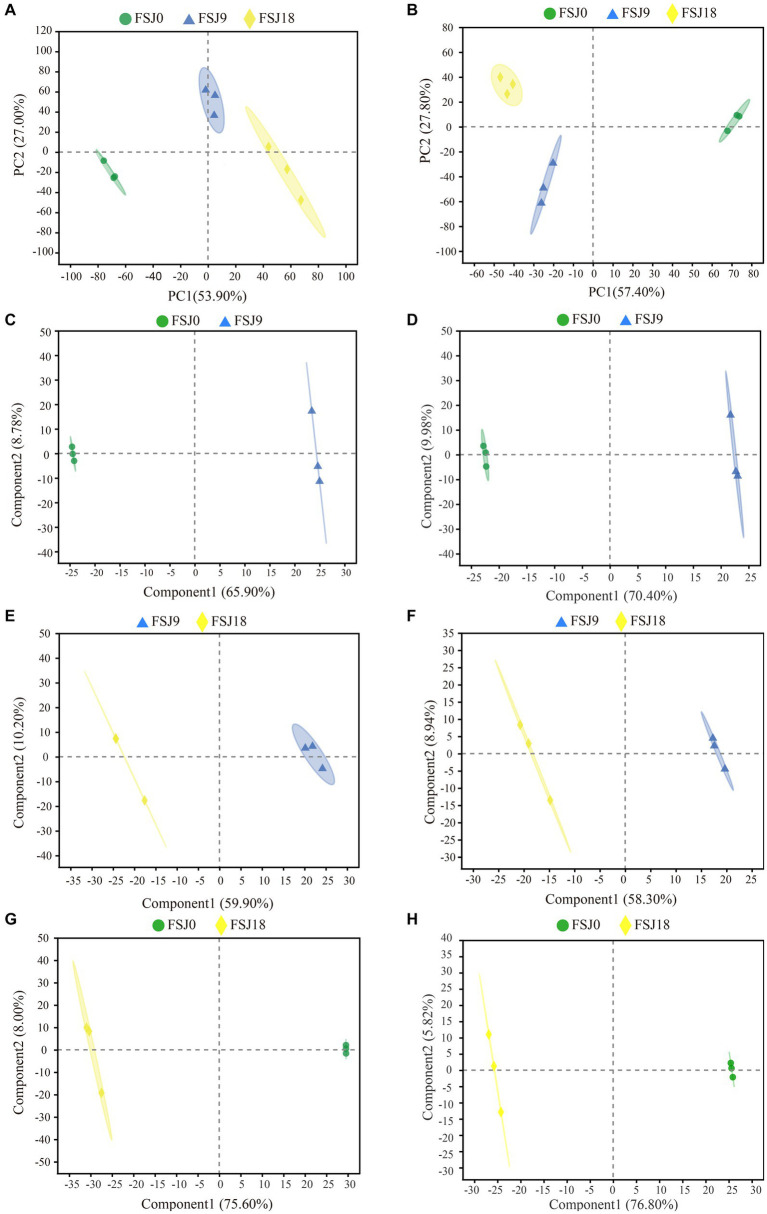
Principal component analysis in positive **(A)** and negative **(B)** ion modes during fermentation. PLS-DA score chart in positive **(C, E, G)** and negative **(D, F, H)** ion modes during fermentation.

In order to gain a better understanding of the metabolic changes in yeast-fermented sea buckthorn beverages during the fermentation process, PCA was conducted on the metabolites collected in both the positive and negative ion modes. As shown in Figure 3A, in the positive ion mode, the first and second principal components (PC1 and P2) accounted for 53.90 and 27.00% of the total variance, respectively. Along PC1, FSJ0 was positioned on the negative axis, while FSJ18 was positioned on the positive axis. For PC2, FSJ9 and FSJ18 were located on the negative axis, while FSJ9 was positioned on the positive axis of PC1. Additionally, samples FSJ0 and FSJ18 exhibited inter-group differences along PC1 and intra-group clustering along PC2, indicating that the metabolic differences between FSJ0 and FSJ18 could primarily be attributed to the first principal component. Meanwhile, the differences between FSJ0 and FSJ9 and between FSJ9 and FSJ18 were mainly reflected in the second principal component. As illustrated in Figure 3B, in the negative ion mode, PC1 and PC2 accounted for 54.70 and 27.80% of the total variance, respectively. The data points of the three groups showed distinct separation on the PCA score plot, indicating the reliability of this model and the significant variations in non-volatile metabolites during the fermentation process (35).

We further validated the constructed model using the PLS-DA method. The PLS-DA models for the three groups of samples are shown in Figures 3C–G. In both the positive and negative ion modes, FSJ0 was distributed on the left side, while FSJ9 and FSJ18 were distributed on the right side. This indicated that the model could effectively differentiate FSJ0 from FSJ9 and FSJ18, demonstrating the clear differences in metabolite species and levels between FSJ0, and FSJ9 and FSJ18. However, it should be noted that in PLS-DA modeling, the samples are specified and grouped, which can lead to overfitting of the data. To address this concern, a random permutation test was performed for external cross-validation of the model. The results are shown in Table 3. In both the positive and negative ion modes, the cross-validation parameters R2X, R2Y, and Q2 of the PLS-DA model were greater than 0.5, indicating that the model had good explanatory and predictive capabilities and there was no evidence of overfitting. Therefore, the PLS-DA model established in this study exhibited good predictive performance and high predictive ability, effectively explaining the metabolic differences among the three sample groups (FSJ0, FSJ9, and FSJ18) (36).

**Table 3:** Evaluation parameters of the PLS-DA model.

Group	Positive ion mode			Negative ion mode		
	R^2^X	R^2^Y	Q^2^	R^2^X	R^2^Y	Q^2^
FSJ0 versus FSJ9	0.659	1	0.984	0.704	1	0.989
FSJ9 versus FSJ18	0.699	0.987	0.955	0.683	0.989	0.955
FSJ0 versus FSJ18	0.756	0.999	0.992	0.768	0.999	0.993

#### 3.2.3. Screening and identification of key differential metabolites

Based on the results of PLS-DA, VIP values were utilized as the screening criterion for identifying differential metabolites (37). A VIP cutoff of >2 was applied to identify metabolites showing significant differences across different stages of fermentation in sea buckthorn juice. A total of 60 differential metabolites (in both the positive and negative ion modes) were identified in the FSJ0 versus FSJ9, FSJ9 versus FSJ18, and FSJ0 versus FSJ18 comparison groups. Detailed results are shown in Supplementary Tables S2–S4.

**Figure 4:**
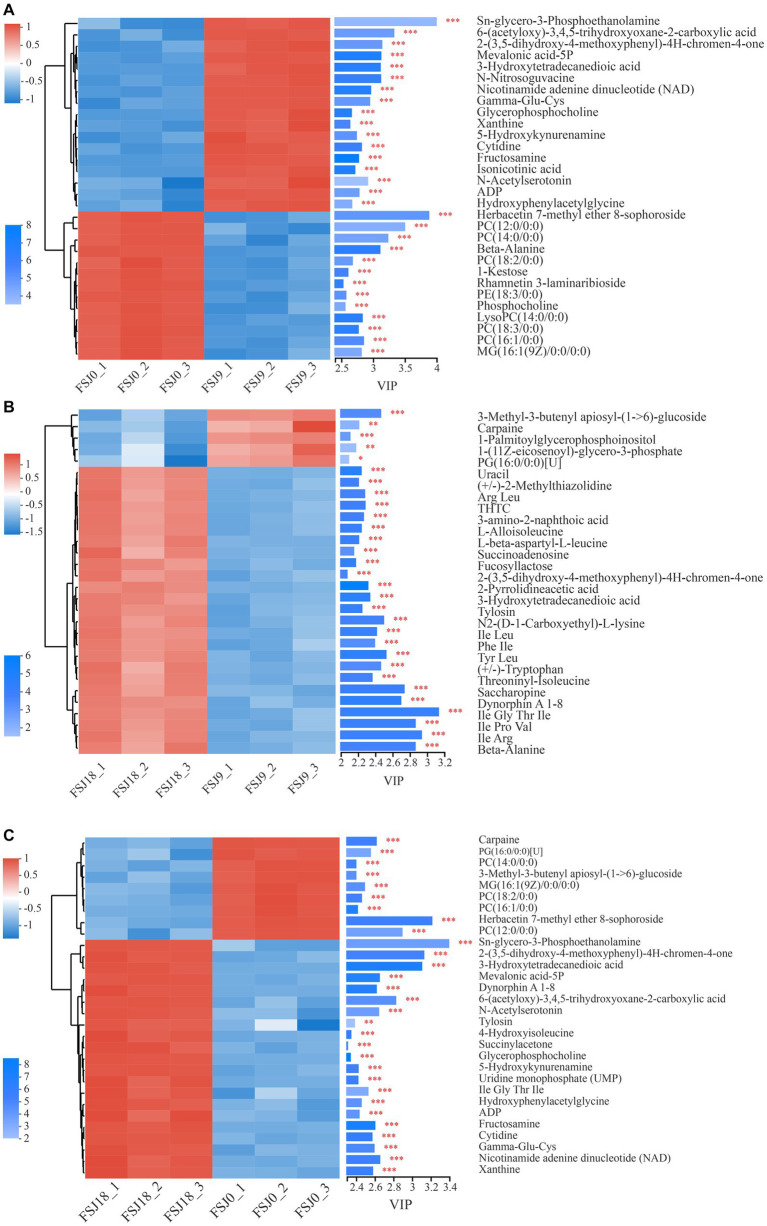
Analysis of differential metabolites in the FSJ0 versus FSJ9 **(A)**, FSJ9 versus FSJ18 **(B)**, and FSJ0 versus FSJ18 **(C)** (VIP > 2).

As shown in Figure 4A, a total of 30 differential metabolites were identified in yeast-fermented sea buckthorn beverages at 0 h versus 9 h of fermentation. Among them, 17 metabolites showed upregulation, while 13 showed downregulation. Glycerophosphocholine and LysoPC (14:0/0:0), which are involved in glycerophospholipid metabolism and choline metabolism, were identified as differential metabolites. The content of glycerophosphocholine increased from 5.65 ± 0.01 at 0 h of fermentation to 7.00 ± 0.04 (*p* < 0.05) at 9 h of fermentation, while that of LysoPC (14, 0/0, 0) decreased from 4.76 ± 0.03 to 3.16 ± 0.04 (*p* < 0.05). The content of Gamma-Glu-Cys and hydroxyphenylacetylglycine — which are related to amino acid metabolism —increased from 3.00 ± 0.12 to 4.67 ± 0.01 (*p* < 0.05) and from 2.55 ± 0.14 to 3.93 ± 0.03 (*p* < 0.05), respectively. Moreover, the content of isonicotinic acid, N-acetylserotonin, and xanthine — all of which are involved in the biosynthesis of secondary metabolites along with 1-kestose and beta-alanine — increased from 3.66 ± 0.02 to 5.08 ± 0.03 (*p* < 0.05), from 1.94 ± 0.23 to 3.59 ± 0.08 (*p* < 0.05), and from 2.83 ± 0.05 to 4.21 ± 0.04 (*p* < 0.05), respectively.

As shown in Figure 4B, a total of 30 different metabolites (26 upregulated and 4 downregulated) were identified in the yeast-fermented sea buckthorn beverage at 9 h versus 18 h of fermentation. The metabolites showing downregulation included carpaine, 3-methyl-3-butenyl apiosyl-(1- > 6)-glucoside, and 1-palmitoylglycerophosphoinositol. Carpaine is a bioactive alkaloid with anti-platelet depletion activity, and it can maintain platelet count and exert anti-malarial effects. Its content decreased from 3.11 ± 0.18 at 9 h to 2.44 ± 0.09 (*p* < 0.05) at 18 h. In contrast, the metabolites exhibiting an upregulated trend included L-alloisoleucine, beta-alanine, saccharopine, and (+/−)-tryptophan. Saccharopine is an intermediate in lysine degradation, and it is converted to saccharopine via lysine-ketoglutarate reductase. Saccharopine is further oxidized to α-aminoadipic semialdehyde and glutamate by saccharopine dehydrogenase (38). The content of saccharopine increased from 4.26 ± 0.06 at 9 h to 5.24 ± 0.09 (*p* < 0.05) at 18 h. (+/−)-Tryptophan is an aromatic amino acid used as a nutritional supplement and can treat niacin deficiency; its content increased from 3.42 ± 0.03 at 9 h to 4.22 ± 0.12 (*p* < 0.05) at 18 h.

As shown in Figure 4C, a total of 30 different metabolites were identified in the yeast-fermented sea buckthorn beverage at 0 h versus 18 h of fermentation, with 21 metabolites being upregulated and 9 being downregulated. The metabolites showing downregulation included carpaine, PG (16, 0/0, 0), phosphatidylcholine (PC; 14, 0/0, 0), PC (18, 2/0, 0), PC (16, 1/0, 0), and 3-methyl-3-butenyl apiosyl-(1- > 6)-glucoside. Meanwhile, Gamma-Glu-Cys, dynorphin A 1–8, ADP, cytidine, glycerophosphocholine, and Sn-glycero-3-phosphoethanolamine were upregulated. Glycerophosphocholine participates in glycerophospholipid metabolism and choline metabolism, and its content increased from 5.65 ± 0.01 at 0 h to 7.19 ± 0.01 (*p* < 0.05) at 18 h. Glycerophosphocholine is enzymatically broken down into choline and glycerophospholipids; choline is involved in acetylcholine biosynthesis and glycerophospholipids are involved in phosphatidylcholine synthesis. Glycerophosphocholine plays a role in the metabolism of choline, aiding in the synthesis of acetylcholine and phosphatidylcholine, and greatly contributes to neurocognitive function and memory (39). Xanthine is a purine alkaloid present in human organs and bodily fluids. It is used as a mild stimulant and bronchodilator for treating asthma symptoms and thus has a certain pharmacological value. Its content increased from 2.83 ± 0.05 at 0 h to 4.55 ± 0.06 (*p* < 0.05) at 18 h in the sea buckthorn beverage (40).

The examination of differential metabolites revealed that the fermentation of sea buckthorn beverages by yeast mainly leads to the generation of lipids and secondary metabolites. Lipids were found to be the most valuable bioactive component in fermented sea buckthorn beverages (41–43).

#### 3.2.4. Analysis of the main metabolites during fermentation

**Figure 5:**
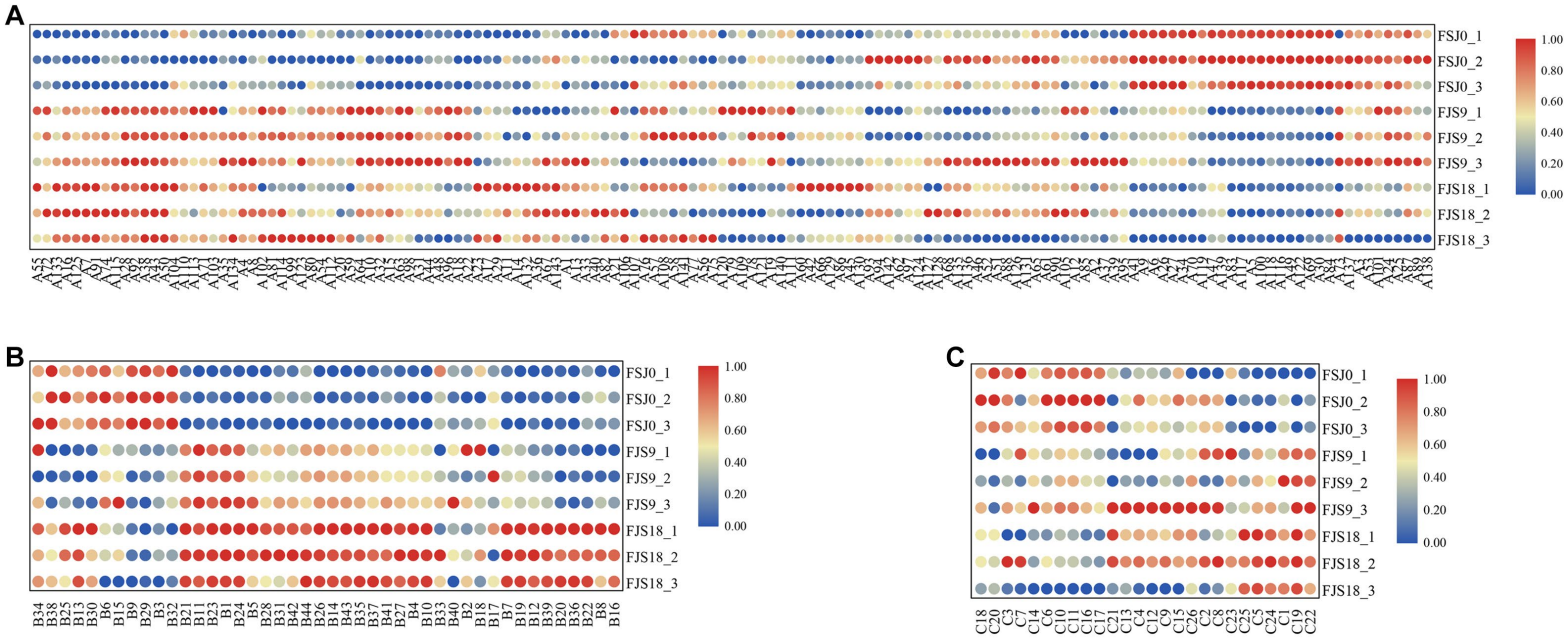
Content changes of lipids and lipid-like molecules **(A)**, amino acids, peptides, and analogues **(B)**, benzenoids **(C)** during fermentation.

Lipids play a crucial role in various cellular activities, including cell development and differentiation, intracellular substance transport, signal transduction, and apoptosis (44). Fatty acids are essential for the functioning of the human body and have various biological activities, including antioxidant and cholesterol-lowering functions (45). The changes in lipid and phospholipid molecules during the fermentation of sea buckthorn beverages by yeast are presented in the Supplementary Table S5. Figure 5A shows that a total of 143 lipid and phospholipid molecules were detected during the yeast fermentation of sea buckthorn beverages. Among them, 80 showed an upward trend, 43 showed a downward trend, and 20 showed relatively stable levels before and after fermentation. The total content of lipids and phospholipids decreased from 700.96 to 695.89 (*p* < 0.05), with a total decrease of 5.07. Compounds such as 1-palmitoyl-sn-glycero-3-phosphocholine [LysoPC (16: 0)], 1-myristoyl-2-hydroxy-sn-glycero-3-phosphocholine [LysoPC (14: 0/0: 0)], and ethyl 2-furanacrylate were reduced after fermentation, with their content decreasing by 1.00, 1.37, and 0.37, respectively. Among them, LysoPC (16: 0) and LysoPC (14: 0/0: 0) are involved in glycerophospholipid metabolism, and LysoPC (14, 0/0, 0) is a hemolytic phospholipid with effective anti-spasmodic properties. After fermentation, the content of Sn-glycero-3-phosphoethanolamine, adipic acid, glycerophosphocholine, kojibiose, 3-oxohexadecanoic acid, 3-hydroxytetradecanedioic acid, 2-hydroxyhexadecanoic acid, tanacetol A, 2-isopropylmalic acid, and methyl 3-(methylthio) butanoate increased. Among them, LysoPC (16, 0) and LysoPC (18: 1(9Z)) are important substrates for the synthesis of PC (46). Glycerophosphocholine is an important biological precursor of acetylcholine, a key neurotransmitter with a significant role in the nervous system. Glycerophosphocholine has been shown to enhance cognitive abilities and memory in older individuals and holds significant application value in the food and pharmaceutical industries (39). Interestingly, the content of glycerophosphocholine also increased in sea buckthorn juice after yeast fermentation. This could be attributed to the generation of glycerophosphocholine from LysoPC under the catalytic action of hemolytic phospholipase I during fermentation (47).

Amino acids are fundamental components of proteins in living organisms and are closely associated with various life processes (48, 49). Therefore, they are indispensable nutritional elements. Additionally, amino acids play a crucial role in determining product flavor (50). The changes in amino acids and their derivatives during the fermentation of sea buckthorn beverages by yeast are presented in the Supplementary Table S6. As shown in Figure 5B, a total of 44 amino acids, peptides, and analogs were identified in yeast-fermented sea buckthorn beverages. Among them, 32 showed an upward trend, while 12 showed a downward trend following fermentation.

L-asparagine plays a crucial role in maintaining the structure and function of proteins. It can undergo glycosylation by reacting with N-acetylglucosamine under the action of oligosaccharide transferase (51). During fermentation, its concentration decreased from 6.18 ± 0.02 to 5.73 ± 0.01 (*p* < 0.05). L-Tyrosine, one of the three major aromatic amino acids, is a precursor for various compounds involved in maintaining human health and metabolism, such as thyroid hormones, melanin (52), and dopamine (53). There was no significant change in its content during fermentation. L-Homoserine, an important intermediate for methionine, threonine, and lysine, showed an increase in concentration from 4.78 ± 0.01 to 5.11 ± 0.03 (*p* < 0.05) during fermentation, reaching levels as high as 5.4 ± 0.02. Gamma-Glu-Cys, a dipeptide composed of cysteine and glutamic acid and a precursor of glutathione, increased in concentration from 3.00 ± 0.12 to 4.67 ± 0.01 (*p* < 0.05), ultimately reaching levels as high as 4.91 ± 0.05. Glutathione, a tripeptide compound composed of glutamic acid, cysteine, and glycine, acts as a primary antioxidant and counteracts free radical damage in the human body, providing protection to the liver (54). Its concentration increased from 4.93 ± 0.01 to 6.42 ± 0.04 (*p* < 0.05) during fermentation. N-Acetyl-L-glutamic acid, involved in arginine biosynthesis and urea cycle regulation, showed an increase in concentration from 3.29 ± 0.03 to 3.88 ± 0.02 (*p* < 0.05), ultimately reaching a level of 4.09 ± 0.05. L-Serine, a precursor for phospholipids, amino acids, and proteins, participates in the synthesis of glycine and cysteine through transsulfuration and contributes to glutathione generation (55). Glutathione possesses antioxidant, free-radical scavenging, and immune-enhancing properties (54). Additionally, it imparts a robust flavor and enhances sweetness in food products (56). The levels of L-serine and glutathione increased after 18 h of fermentation in yeast-fermented sea buckthorn beverages, positively impacting their antioxidant capacity and taste.

Based on Figure 5C, it can be observed that a total of 26 benzenoid compounds were detected during the fermentation process, with 12 exhibiting an increasing trend and 14 showing a decreasing trend. Among them, benzylformate — which exhibits a strong floral aroma with hints of fruit and European blackberry — showed relatively stable levels throughout fermentation (ranging from 3.71 ± 0.02 to 3.72 ± 0.05) (15). 4-Ethylphenol, a compound with a pleasant aroma that is used in coffee and other fragrances — also showed a relatively stable content after fermentation (varying from 4.19 ± 0.02 to 4.18 ± 0.03). The content of phenylacetaldehyde, which emits a floral scent reminiscent of hyacinths with undertones of almond and cherry fragrance (15), was 4.88 ± 0.02. Another key compound detected was vanillin, which possesses a strong fragrance of vanilla pods. Its content increased from 3.51 ± 0.03 to 3.55 ± 0.03 (*p* < 0.05) during the fermentation process, ultimately reaching 3.70 ± 0.03. Vanillin has aromatic, antimicrobial, and antioxidant properties, imparting a rich milky aroma to sea buckthorn juice and positively impacting the quality of yeast-fermented sea buckthorn beverages (57).

#### 3.2.5. Metabolite enrichment pathways in yeast-fermented sea buckthorn beverages

**Figure 6:**
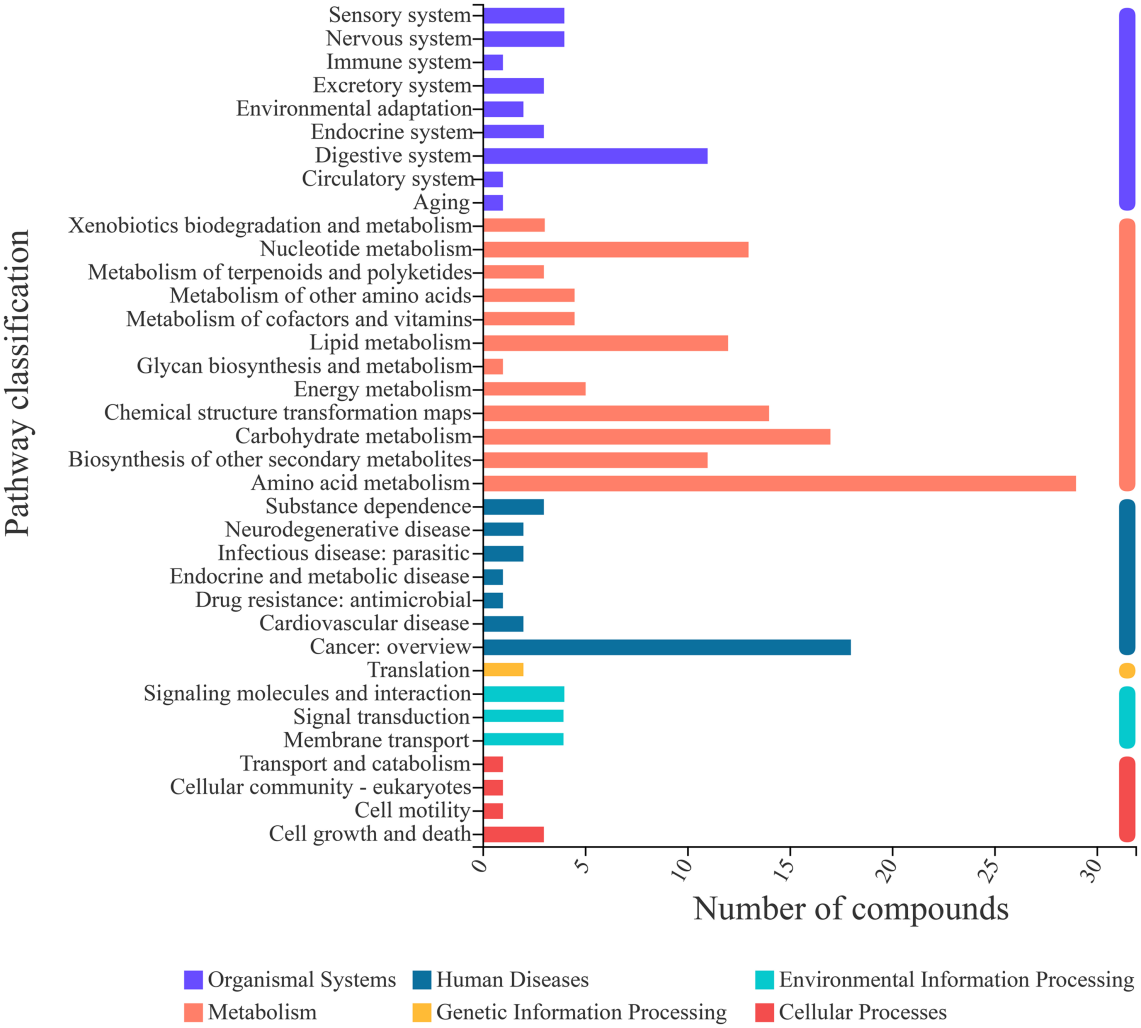
KEGG metabolite statistics.

Fermentation is a complex process; thus, an analysis of the metabolic pathways involved in fermentation is warranted. Through comparison with the KEGG database, metabolic pathway information was obtained for the metabolites identified in this study (Figure 6). A total of 36 metabolic pathways belonging to 6 categories were involved in the fermentation of sea buckthorn beverages. The pathways included amino acid metabolism (29 metabolites), carbohydrate metabolism (19 metabolites), nucleotide metabolism (14 metabolites), chemical structure transformation maps (14 metabolites), lipid metabolism (13 metabolites), biosynthesis of other secondary metabolites (12 metabolites), and digestive system (10 metabolites). Each of these pathways involved more than 10 metabolites. Hence, these were the main metabolic pathways involved in the yeast fermentation of sea buckthorn beverages.

## 4. Conclusion

Based on our analysis, the following conclusions can be drawn: (1) Sea buckthorn juice and fermented sea buckthorn juice predominantly contain esters, alcohols, aldehydes, acids, and ketones. The aromatic properties of unfermented sea buckthorn juice are largely influenced by esters, aldehydes, and acids. Meanwhile, fermented sea buckthorn juice is characterized by the presence of esters, aldehydes, and ketones. (2) Yeast fermentation leads to a decrease in the presence of compounds associated with a rancid odor, resulting in an overall improvement in the flavor of sea buckthorn juice. Furthermore, it increases the concentration of esters and alcohols, which confer fruity and floral aromas to the juice. (3) A total of 379 metabolites were detected in yeast-fermented sea buckthorn beverages at 0 h, 9 h, and 18 h, and 60 differential metabolites were identified and analyzed (VIP > 2). (4) The relative content of bioactive components, such as glycerophosphorylcholine, glutathione, L-glutamic acid, and vanillin, increased in sea buckthorn juice through fermentation. (5) Amino acid metabolism and carbohydrate metabolism were found to be the primary metabolic pathways active during the fermentation of sea buckthorn beverages. Thus, the flavor of sea buckthorn juice was improved by yeast fermentation, and its bioactive content was increased. This study provides information at the molecular level for quality control and optimisation of the yeast fermentation process of sea buckthorn juice. In the future, studies evaluating the effects of bioactive components on the functional properties of fermented sea buckthorn juice are warranted.

## Data availability statement

The original contributions presented in the study are included in the article/Supplementary material, further inquiries can be directed to the corresponding author.

## Author contributions

BP: Writing – original draft, Data curation, Formal analysis, Software, Visualization, Writing – review & editing. JL: Data curation, Formal analysis, Software, Writing – review & editing. CS: Conceptualization, Funding acquisition, Supervision, Writing – review & editing. WC: Data curation, Writing – review & editing. QZ: Methodology, Validation, Writing – review & editing. XZ: Supervision, Writing – review & editing. SL: Writing – review & editing. JW: Writing – review & editing. LJ: Writing – review & editing. XY: Resources, Writing – review & editing. FT: Methodology, Project administration, Writing – review & editing. All authors contributed to the article and approved the submitted version.

## Funding

The author(s) declare financial support was received for the research, authorship, and/or publication of this article. This work was supported by the Science and Technology Innovation Talent Program of Xinjiang Production and Construction Corps (2020CB025); and the Projects of Innovation and Development Pillar Program for Key Industries in Southern Xinjiang of Xinjiang Production and Construction Corps (2022DB007); and Science and Technology Program Projects in the Sixth Division of Wujiaqu City (2315).

## Conflict of interest

The authors declare that the research was conducted in the absence of any commercial or financial relationships that could be construed as a potential conflict of interest.

## Publisher’s note

All claims expressed in this article are solely those of the authors and do not necessarily represent those of their affiliated organizations, or those of the publisher, the editors and the reviewers. Any product that may be evaluated in this article, or claim that may be made by its manufacturer, is not guaranteed or endorsed by thepublisher.
