# Supplementary material for: Exploring metabolic dynamics during the fermentation of sea buckthorn beverage: comparative analysis of volatile aroma compounds and non-volatile metabolites using GC–MS and UHPLC–MS
Source: Front Nutr. 2023 Sep 7;10:1268633. doi: 10.3389/fnut.2023.1268633 (PMC10512423; doi:10.3389/fnut.2023.1268633)
Supplement: Supplementary file 1 [file Data_Sheet_1.docx]

**Supplementary Table 1** Changes of metabolite during the fermentation process

| Code | M/Z | Mode | Metabolite | Content | | |
| --- | --- | --- | --- | --- | --- | --- |
|  |  |  |  | 0 h | 9 h | 18 h |
| F1 | 153.02 | neg | Gentisic acid | 3.68±0.02 | 3.79±0.01 | 3.76±0.01 |
| F2 | 325.18 | neg | 2-Dodecylbenzenesulfonic acid | 5.60±0.08 | 5.70±0.08 | 5.67±0.04 |
| F3 | 165.05 | neg | Atrolactic acid | 4.64±0.01 | 4.79±0.04 | 4.89±0.03 |
| F4 | 151.04 | neg | Vanillin | 3.51±0.03 | 3.55±0.03 | 3.70±0.03 |
| F5 | 405.18 | neg | (4-Ethoxyphenyl)urea | 4.26±0.04 | 4.31±0.03 | 4.29±0.02 |
| F6 | 279.16 | pos | Phthalic acid Mono-2-ethylhexyl Ester | 5.31±0.02 | 5.35±0.03 | 5.36±0.01 |
| F7 | 135.08 | pos | 2,5-Dimethylbenzaldehyde | 4.96±0.05 | 4.98±0.05 | 4.95±0.05 |
| F8 | 121.06 | pos | P-Tolualdehyde | 4.88±0.01 | 4.84±0.02 | 4.83±0.07 |
| F9 | 231.11 | pos | Fenamic acid | 5.56±0.03 | 5.54±0.07 | 5.55±0.06 |
| F10 | 308.09 | pos | Avenanthramide L | 4.17±0.05 | 5.03±0.01 | 5.43±0.05 |
| F11 | 176.07 | pos | 4-Hydroxy-5-phenyltetrahydro-1,3-oxazin-2-one | 4.45±0.02 | 4.39±0.03 | 4.35±0.04 |
| F12 | 325.25 | pos | Dihydrocapsaicin | 4.59±0.37 | 4.54±0.34 | 4.38±0.47 |
| F13 | 119.09 | pos | Alpha-Methylstyrene | 5.66±0.04 | 5.69±0.06 | 5.67±0.05 |
| F14 | 137.06 | pos | Benzylformate | 3.71±0.02 | 3.74±0.04 | 3.72±0.05 |
| F15 | 137.10 | pos | 4-Propylphenol | 4.37±0.02 | 4.28±0.04 | 4.14±0.07 |
| F16 | 133.10 | pos | P-Mentha-1,3,5,8-tetraene | 4.66±0.03 | 4.57±0.04 | 4.41±0.08 |
| F17 | 211.10 | pos | Sinapyl alcohol | 4.54±0.01 | 4.53±0.07 | 4.54±0.05 |
| F18 | 236.13 | pos | Butylparaben | 4.12±0.02 | 4.13±0.08 | 4.15±0.03 |
| F19 | 170.10 | pos | Diphenylamine | 4.16±0.01 | 4.19±0.04 | 4.10±0.02 |
| F20 | 123.08 | pos | 4-Ethylphenol | 4.19±0.02 | 4.19±0.03 | 4.18±0.03 |
| F21 | 147.04 | pos | Phenylpyruvic acid | 4.97±0.03 | 4.76±0.06 | 4.59±0.07 |
| F22 | 119.05 | pos | Phenylacetic acid | 3.73±0.06 | 3.52±0.05 | 3.38±0.05 |
| F23 | 144.08 | pos | 2-Naphthylamine | 4.35±0.04 | 4.23±0.08 | 4.26±0.04 |
| F24 | 236.13 | pos | Bk-DMBDB | 3.54±0.00 | 4.39±0.06 | 4.43±0.04 |
| F25 | 107.05 | pos | Benzaldehyde | 4.50±0.06 | 3.96±0.03 | 4.19±0.05 |
| F26 | 121.06 | pos | Phenylacetaldehyde | 4.86±0.01 | 4.87±0.03 | 4.88±0.02 |
| G1 | 161.04 | neg | 3-hydroxy-3-methyl-Glutaric acid | 4.59±0.03 | 4.59±0.02 | 4.63±0.02 |
| G2 | 337.11 | neg | Pantoyllactone glucoside | 4.12±0.01 | 4.14±0.02 | 4.13±0.02 |
| G3 | 329.23 | neg | 9,10,13-TriHOME | 4.35±0.02 | 4.40±0.06 | 4.42±0.06 |
| G4 | 487.34 | neg | 4a-Carboxy-4b-methyl-5a-cholesta-8,24-dien-3b-ol | 4.76±0.02 | 4.78±0.05 | 4.86±0.04 |
| G5 | 297.24 | neg | Cis-9,10-Epoxystearic acid | 4.41±0.01 | 4.72±0.06 | 4.64±0.08 |
| G6 | 269.21 | neg | 3-Oxohexadecanoic acid | 4.22±0.01 | 4.70±0.02 | 4.79±0.06 |
| G7 | 617.39 | neg | 3-O-cis-Coumaroylmaslinic acid | 4.58±0.03 | 4.71±0.08 | 4.83±0.09 |
| G8 | 571.29 | neg | 1-Palmitoylglycerophosphoinositol | 5.33±0.02 | 6.09±0.03 | 5.49±0.11 |
| G9 | 509.29 | neg | 1-(11Z-eicosenoyl)-glycero-3-phosphate | 3.95±0.02 | 4.36±0.13 | 3.68±0.21 |
| G10 | 175.06 | neg | 2-Isopropylmalic acid | 4.47±0.01 | 4.94±0.03 | 5.14±0.04 |
| G11 | 775.53 | neg | PA(18:1(9Z)/22:2(13Z,16Z)) | 4.46±0.03 | 4.57±0.06 | 4.50±0.06 |
| G12 | 517.23 | neg | 6S,9R-Dihydroxy-4,7E-megastigmadien-3-one 9-[apiosyl-(1->6)-glucoside] | 4.84±0.02 | 4.86±0.04 | 4.88±0.02 |
| G13 | 339.20 | neg | NORETHINDRONE ACETATE | 5.34±0.07 | 5.39±0.06 | 5.39±0.05 |
| G14 | 477.22 | neg | Prostaglandin G2 2-glyceryl Ester | 4.01±0.00 | 4.62±0.10 | 4.20±0.08 |
| G15 | 523.34 | neg | Ganoderiol I | 4.18±0.04 | 4.21±0.05 | 4.49±0.16 |
| G16 | 431.19 | neg | Corchoionol C 9-glucoside | 5.10±0.02 | 5.12±0.02 | 5.09±0.01 |
| G17 | 433.24 | neg | LysoPA(0:0/18:2(9Z,12Z)) | 5.11±0.02 | 5.14±0.03 | 4.69±0.09 |
| G18 | 407.22 | neg | 1-(9Z-hexadecenoyl)-glycero-3-phosphate | 5.10±0.03 | 5.18±0.05 | 4.72±0.07 |
| G19 | 480.31 | neg | LysoPC(15:0) | 5.29±0.02 | 4.59±0.03 | 4.21±0.14 |
| G20 | 452.28 | neg | PE(16:0/0:0) | 5.44±0.03 | 5.19±0.01 | 4.92±0.09 |
| G21 | 309.12 | neg | (Z)-2-Methyl-2-butene-1,4-diol 4-O-beta-D-Glucopyranoside | 5.13±0.02 | 5.12±0.03 | 5.06±0.03 |
| G22 | 271.23 | neg | 2-hydroxyhexadecanoic acid | 3.58±0.02 | 4.62±0.07 | 4.55±0.08 |
| G23 | 471.35 | neg | Azukisapogenol | 4.60±0.03 | 4.77±0.05 | 5.03±0.12 |
| G24 | 512.30 | neg | LysoPC(14:0/0:0) | 4.76±0.03 | 3.16±0.04 | 3.39±0.06 |
| G25 | 441.20 | neg | 1-Hexanol arabinosylglucoside | 5.03±0.02 | 5.11±0.02 | 5.10±0.01 |
| G26 | 269.21 | neg | 5,10-Pentadecadien-1-ol | 3.86±0.01 | 4.12±0.10 | 3.98±0.10 |
| G27 | 293.18 | neg | Furanofukinin | 4.11±0.00 | 4.14±0.00 | 4.13±0.00 |
| G28 | 327.22 | neg | Corchorifatty acid F | 4.04±0.04 | 4.09±0.06 | 4.08±0.04 |
| G29 | 379.16 | neg | 3-Methyl-3-butenyl apiosyl-(1->6)-glucoside | 4.28±0.04 | 3.58±0.03 | 2.78±0.12 |
| G30 | 477.10 | neg | Isorhamnetin 3-glucoside | 6.47±0.03 | 6.57±0.04 | 6.56±0.02 |
| G31 | 351.13 | neg | 4-Ethoxy-4-oxobutanoic acid | 4.62±0.01 | 4.66±0.05 | 4.66±0.01 |
| G32 | 451.22 | neg | (R)-1-O-[b-D-Glucopyranosyl-(1->6)-b-D-glucopyranoside]-1,3-octanediol | 4.05±0.02 | 4.07±0.06 | 4.15±0.02 |
| G33 | 293.12 | neg | Ethyl (S)-3-hydroxybutyrate glucoside | 4.53±0.02 | 4.54±0.03 | 4.53±0.02 |
| G34 | 145.05 | neg | Adipic acid | 3.65±0.17 | 4.67±0.05 | 4.73±0.02 |
| G35 | 309.12 | neg | Ilicifolinoside A | 4.18±0.02 | 4.18±0.03 | 4.18±0.01 |
| G36 | 365.11 | neg | 1-O-alpha-D-Glucopyranosyl-D-mannitol | 5.25±0.01 | 5.27±0.00 | 5.29±0.02 |
| G37 | 566.35 | neg | LysoPC(18:1(9Z)) | 5.95±0.02 | 5.37±0.06 | 4.99±0.10 |
| G38 | 538.32 | neg | LysoPC(16:1(9Z)/0:0) | 6.14±0.04 | 4.98±0.07 | 4.91±0.05 |
| G39 | 540.33 | neg | LysoPC(16:0) | 6.21±0.02 | 5.55±0.03 | 5.21±0.11 |
| G40 | 333.06 | neg | 1-(sn-Glycero-3-phospho)-1D-myo-inositol | 5.69±0.01 | 6.47±0.01 | 6.24±0.03 |
| G41 | 496.34 | pos | PC(16:0/0:0) | 6.93±0.03 | 5.97±0.10 | 5.56±0.11 |
| G42 | 223.13 | pos | Dehydrovomifoliol | 4.46±0.02 | 4.52±0.04 | 4.38±0.03 |
| G43 | 170.15 | pos | (S)-(-)-Perillyl alcohol | 5.04±0.04 | 5.09±0.01 | 5.08±0.04 |
| G44 | 170.15 | pos | (R)-Carvotanacetone | 4.88±0.02 | 4.96±0.01 | 4.94±0.05 |
| G45 | 237.22 | pos | (E)-11-Hexadecenoic acid | 5.28±0.01 | 5.30±0.01 | 5.32±0.02 |
| G46 | 452.28 | pos | LysoPE(16:1(9Z)/0:0) | 5.72±0.04 | 4.86±0.07 | 4.79±0.01 |
| G47 | 468.31 | pos | PC(14:0/0:0) | 5.38±0.04 | 3.37±0.17 | 3.75±0.08 |
| G48 | 281.14 | pos | 8-Deoxy-11,13-dihydroxygrosheimin | 5.34±0.05 | 5.38±0.06 | 5.37±0.05 |
| G49 | 95.09 | pos | 2,5-Heptadien-1-ol | 4.13±0.01 | 4.15±0.00 | 4.15±0.04 |
| G50 | 258.11 | pos | Glycerophosphocholine | 5.65±0.01 | 7.00±0.04 | 7.19±0.01 |
| G51 | 219.14 | pos | 2-(3-Hydroxy-4-methylphenyl)-5-methyl-4-hexen-3-one | 4.13±0.02 | 4.13±0.01 | 4.09±0.04 |
| G52 | 209.15 | pos | 4,5-Dihydrovomifoliol | 4.48±0.02 | 4.49±0.04 | 4.48±0.04 |
| G53 | 197.08 | pos | (S)-Batatic acid | 5.06±0.02 | 5.05±0.06 | 5.05±0.06 |
| G54 | 419.15 | pos | 1-(3-Methylbutanoyl)-6-apiosylglucose | 5.70±0.04 | 5.66±0.06 | 5.66±0.04 |
| G55 | 265.14 | pos | (+)-Abscisic Acid | 4.81±0.03 | 4.81±0.04 | 4.77±0.04 |
| G56 | 181.12 | pos | Norecasantalic acid | 5.09±0.01 | 5.06±0.06 | 4.94±0.09 |
| G57 | 197.08 | pos | Glycerol 1-propanoate diacetate | 3.80±0.02 | 3.79±0.04 | 3.77±0.07 |
| G58 | 319.15 | pos | 3-Hydroxytetradecanedioic acid | 2.05±0.01 | 3.91±0.05 | 4.77±0.05 |
| G59 | 212.20 | pos | Beta-Ionol | 5.21±0.03 | 5.27±0.01 | 5.26±0.05 |
| G60 | 256.23 | pos | Germacrenone | 4.73±0.02 | 4.79±0.02 | 4.80±0.08 |
| G61 | 325.11 | pos | Kojibiose | 4.41±0.05 | 5.11±0.05 | 5.00±0.04 |
| G62 | 129.05 | pos | 3-Methylglutaric acid | 5.72±0.00 | 5.75±0.01 | 5.75±0.01 |
| G63 | 638.57 | pos | DG(18:0/18:2(9Z,12Z)/0:0) | 6.01±0.02 | 6.02±0.02 | 6.03±0.02 |
| G64 | 315.25 | pos | 9,10-DHOME | 7.41±0.02 | 7.42±0.02 | 7.42±0.02 |
| G65 | 131.09 | pos | 5-Hydroxy-p-mentha-6,8-dien-2-one | 5.22±0.04 | 5.22±0.04 | 5.22±0.03 |
| G66 | 319.15 | pos | S-(2-Methylpropionyl)-dihydrolipoamide-E | 4.53±0.04 | 4.48±0.08 | 4.60±0.02 |
| G67 | 256.26 | pos | Palmitic amide | 6.66±0.02 | 6.74±0.02 | 6.69±0.05 |
| G68 | 262.24 | pos | Ethyl 3-hydroxydodecanoate | 4.13±0.10 | 4.06±0.07 | 4.18±0.03 |
| G69 | 133.10 | pos | 4-Isopropyl-3-cyclohexene-1-carboxylic acid | 4.39±0.01 | 4.41±0.02 | 4.41±0.04 |
| G70 | 246.24 | pos | 1-Hexanol | 4.75±0.08 | 4.62±0.07 | 4.73±0.02 |
| G71 | 177.16 | pos | 10-Undecenyl acetate | 5.15±0.02 | 5.14±0.05 | 5.12±0.06 |
| G72 | 362.33 | pos | 10,20-Dihydroxyeicosanoic acid | 4.97±0.17 | 5.10±0.17 | 5.04±0.01 |
| G73 | 238.04 | pos | Sn-glycero-3-Phosphoethanolamine | 1.93±0.38 | 5.03±0.04 | 5.22±0.02 |
| G74 | 172.17 | pos | (-)-Carvomenthone | 4.75±0.00 | 4.78±0.00 | 4.74±0.00 |
| G75 | 346.33 | pos | Polyoxyethylene 40 monostearate | 4.75±0.11 | 4.69±0.06 | 4.75±0.03 |
| G76 | 342.30 | pos | (13R,14R)-8-Labdene-13,14,15-triol | 4.48±0.02 | 5.23±0.01 | 4.83±0.06 |
| G77 | 320.26 | pos | Butyl dodecanoate | 4.65±0.00 | 4.74±0.03 | 4.74±0.06 |
| G78 | 518.32 | pos | PC(18:3/0:0) | 5.22±0.04 | 3.75±0.03 | 3.74±0.06 |
| G79 | 201.11 | pos | Decenedioic acid | 4.94±0.01 | 4.95±0.01 | 4.92±0.01 |
| G80 | 242.25 | pos | Tridecanol | 4.46±0.02 | 4.54±0.02 | 4.51±0.07 |
| G81 | 479.33 | pos | Polyporusterone A | 5.89±0.01 | 5.93±0.01 | 5.92±0.01 |
| G82 | 109.10 | pos | (3E,5Z)-3,5-Octadien-1-ol | 4.41±0.04 | 4.45±0.00 | 4.45±0.03 |
| G83 | 323.29 | pos | Serratol | 5.23±0.02 | 5.27±0.03 | 5.26±0.05 |
| G84 | 467.19 | pos | Cynaroside A | 5.02±0.04 | 4.99±0.03 | 5.00±0.01 |
| G85 | 441.24 | pos | MG(0:0/22:6(4Z,7Z,10Z,13Z,16Z,19Z)/0:0) | 5.00±0.11 | 4.95±0.05 | 5.16±0.16 |
| G86 | 619.53 | pos | DG(18:0/18:3(6Z,9Z,12Z)/0:0) | 5.32±0.05 | 5.29±0.02 | 5.30±0.02 |
| G87 | 231.16 | pos | Dodecanedioic acid | 4.13±0.07 | 4.06±0.11 | 4.14±0.08 |
| G88 | 203.13 | pos | Sebacic acid | 6.94±0.02 | 6.95±0.02 | 6.95±0.01 |
| G89 | 613.48 | pos | DG(18:4(6Z,9Z,12Z,15Z)/18:2(9Z,12Z)/0:0) | 4.83±0.05 | 4.92±0.08 | 4.75±0.00 |
| G90 | 245.17 | pos | Cucurbic acid | 4.80±0.07 | 4.88±0.04 | 4.84±0.03 |
| G91 | 363.25 | pos | Trans-Dec-2-enoic acid | 5.35±0.01 | 5.35±0.03 | 5.35±0.02 |
| G92 | 335.07 | pos | Methyl 3-(methylthio)butanoate | 4.95±0.02 | 5.72±0.02 | 5.39±0.07 |
| G93 | 335.22 | pos | 8(R)-Hydroperoxylinoleic acid | 5.61±0.01 | 5.66±0.01 | 5.63±0.02 |
| G94 | 240.23 | pos | Humulol | 4.83±0.03 | 4.90±0.01 | 4.88±0.06 |
| G95 | 477.32 | pos | 3,4-Dimethyl-5-pentyl-2-furanpropanoic acid | 5.68±0.01 | 5.72±0.02 | 5.70±0.01 |
| G96 | 123.12 | pos | (3Z,6Z)-3,6-Nonadien-1-ol | 4.39±0.01 | 4.46±0.00 | 4.45±0.04 |
| G97 | 337.23 | pos | 9,10-DiHOME | 5.79±0.01 | 5.84±0.01 | 5.84±0.01 |
| G98 | 337.27 | pos | Yucalexin P21 | 5.65±0.03 | 5.26±0.01 | 5.20±0.02 |
| G99 | 355.28 | pos | MG(18:2(9Z,12Z)/0:0/0:0)[rac] | 5.09±0.04 | 4.35±0.05 | 4.29±0.02 |
| G100 | 329.27 | pos | MG(16:1(9Z)/0:0/0:0) | 5.25±0.04 | 3.73±0.14 | 3.49±0.14 |
| G101 | 329.16 | pos | Nepetaside | 4.58±0.03 | 4.49±0.02 | 4.47±0.03 |
| G102 | 722.50 | pos | PE(16:1(9Z)/P-18:1(9Z)) | 5.40±0.01 | 5.42±0.02 | 5.40±0.01 |
| G103 | 766.53 | pos | PE(16:0/20:2(11Z,14Z)) | 5.49±0.01 | 5.51±0.02 | 5.48±0.01 |
| G104 | 353.27 | pos | MG(0:0/18:3(6Z,9Z,12Z)/0:0) | 5.03±0.04 | 3.85±0.09 | 3.77±0.10 |
| G105 | 320.26 | pos | Palmitic acid | 4.68±0.02 | 4.77±0.05 | 4.74±0.07 |
| G106 | 374.36 | pos | 2(R)-hydroxydocosanoic acid | 4.44±0.05 | 4.37±0.05 | 4.39±0.04 |
| G107 | 277.18 | pos | Tanacetol A | 3.97±0.01 | 4.32±0.06 | 4.46±0.04 |
| G108 | 240.23 | pos | 10-Epijunenol | 4.45±0.03 | 4.45±0.06 | 4.41±0.05 |
| G109 | 418.22 | pos | Melleolide | 5.68±0.04 | 5.65±0.03 | 5.63±0.10 |
| G110 | 105.07 | pos | Ethyl 2-methyl-3,4-pentadienoate | 5.37±0.00 | 5.36±0.03 | 5.37±0.07 |
| G111 | 228.20 | pos | Propyl 2,4-decadienoate | 6.24±0.01 | 6.28±0.01 | 6.29±0.05 |
| G112 | 229.18 | pos | 11-Hydroxy-9-tridecenoic acid | 4.35±0.02 | 4.40±0.01 | 4.43±0.07 |
| G113 | 151.15 | pos | (E)-4,8-Dimethyl-1,3,7-nonatriene | 4.18±0.02 | 4.21±0.01 | 4.23±0.05 |
| G114 | 83.05 | pos | 2-Pentenoic acid | 4.81±0.02 | 4.88±0.01 | 4.83±0.03 |
| G115 | 547.18 | pos | 6-O-Oleuropeoylsucrose | 5.42±0.04 | 5.40±0.06 | 5.36±0.06 |
| G116 | 209.15 | pos | 3-Hydroxy-beta-ionone | 3.91±0.01 | 3.92±0.03 | 4.02±0.02 |
| G117 | 191.14 | pos | Xi-3-(4-Isopropylphenyl)-2-methylpropanal | 3.71±0.03 | 3.87±0.05 | 4.01±0.03 |
| G118 | 282.28 | pos | Oleamide | 4.85±0.15 | 5.15±0.12 | 5.18±0.13 |
| G119 | 218.21 | pos | Dodecanoic acid | 4.50±0.08 | 4.39±0.04 | 4.50±0.03 |
| G120 | 133.05 | pos | Methylsuccinic acid | 4.49±0.03 | 4.46±0.05 | 4.48±0.05 |
| G121 | 149.10 | pos | Cuminaldehyde | 4.24±0.03 | 4.22±0.04 | 4.23±0.03 |
| G122 | 251.15 | pos | Isopentyl beta-D-glucoside | 5.11±0.03 | 5.06±0.05 | 5.05±0.06 |
| G123 | 213.11 | pos | Prenyl glucoside | 3.87±0.04 | 3.79±0.07 | 3.68±0.06 |
| G124 | 247.13 | pos | Tanacetin | 4.45±0.04 | 4.42±0.09 | 4.41±0.06 |
| G125 | 398.18 | pos | 8-Oxodiacetoxyscirpenol | 4.26±0.08 | 4.25±0.06 | 4.18±0.06 |
| G126 | 212.16 | pos | Menthadienyl acetate | 3.70±0.03 | 3.83±0.12 | 3.90±0.29 |
| G127 | 535.19 | pos | Alpha-Crocetin glucosyl ester | 5.24±0.10 | 4.49±0.05 | 4.82±0.07 |
| G128 | 525.23 | pos | 3-Oxo-alpha-ionol 9-[apiosyl-(1->6)-glucoside] | 4.69±0.03 | 4.66±0.13 | 4.75±0.08 |
| G129 | 529.26 | pos | (3b,9R)-5-Megastigmene-3,9-diol 9-[apiosyl-(1->6)-glucoside] | 4.93±0.04 | 4.88±0.08 | 4.90±0.07 |
| G130 | 440.28 | pos | PC(12:0/0:0) | 4.54±0.04 | 2.18±0.25 | 2.15±0.29 |
| G131 | 131.05 | pos | Ethyl 2-furanacrylate | 5.49±0.06 | 4.87±0.03 | 5.12±0.06 |
| G132 | 330.34 | pos | 2-DECENOL | 5.02±0.03 | 4.95±0.01 | 4.92±0.01 |
| G133 | 95.05 | pos | (2E,4E)-2,4-Hexadienoic acid | 5.13±0.02 | 5.20±0.02 | 5.14±0.03 |
| G134 | 465.35 | pos | DG(8:0/15:0/0:0) | 5.87±0.01 | 5.93±0.02 | 5.93±0.01 |
| G135 | 311.22 | pos | (9R,10S,12Z)-9,10-Dihydroxy-8-oxo-12-octadecenoic acid | 5.30±0.01 | 5.36±0.02 | 5.36±0.02 |
| G136 | 732.55 | pos | PC(16:0/16:1(9Z)) | 5.75±0.58 | 6.16±0.04 | 5.76±0.59 |
| G137 | 259.19 | pos | 2-Hydroxypropyl 2-isopropyl-5-methylcyclohexyl carbonate | 5.84±0.01 | 5.87±0.00 | 5.87±0.01 |
| G138 | 732.56 | pos | Araliacerebroside | 5.45±0.08 | 5.48±0.07 | 5.48±0.09 |
| G139 | 245.17 | pos | (+/-)-1,4-Nonanediol diacetate | 5.55±0.06 | 5.56±0.08 | 5.59±0.05 |
| G140 | 617.51 | pos | DG(18:1(9Z)/18:3(6Z,9Z,12Z)/0:0) | 5.14±0.05 | 5.15±0.08 | 5.13±0.07 |
| G141 | 133.06 | pos | Maleamic acid | 6.02±0.02 | 5.68±0.01 | 5.65±0.03 |
| G142 | 589.48 | pos | DG(18:3(6Z,9Z,12Z)/16:1(9Z)/0:0) | 5.06±0.03 | 5.11±0.04 | 5.01±0.01 |
| G143 | 171.10 | pos | Azelaic acid | 4.41±0.02 | 4.50±0.04 | 4.39±0.01 |
| H1 | 278.09 | neg | 1-Methylguanosine | 4.75±0.01 | 4.77±0.00 | 4.79±0.02 |
| H2 | 242.08 | neg | Cytidine | 3.82±0.06 | 5.41±0.03 | 5.54±0.01 |
| H3 | 339.15 | pos | Nicotine glucuronide | 4.82±0.04 | 4.80±0.06 | 4.77±0.05 |
| H4 | 428.04 | pos | ADP | 3.16±0.12 | 4.65±0.01 | 4.84±0.11 |
| H5 | 384.11 | pos | Succinoadenosine | 4.08±0.06 | 4.31±0.01 | 5.06±0.12 |
| H6 | 268.10 | pos | Adenosine | 5.02±0.03 | 6.10±0.01 | 6.47±0.05 |
| H7 | 284.10 | pos | Guanosine | 4.60±0.05 | 4.89±0.04 | 5.01±0.04 |
| J1 | 131.04 | neg | L-Asparagine | 6.18±0.02 | 5.77±0.04 | 5.73±0.01 |
| J2 | 277.12 | neg | Pantetheine | 3.12±0.01 | 3.32±0.07 | 3.84±0.04 |
| J3 | 147.03 | neg | D-2-Hydroxyglutaric acid | 4.16±0.01 | 5.15±0.02 | 5.14±0.00 |
| J4 | 133.01 | neg | Malic acid | 6.93±0.01 | 6.93±0.01 | 6.87±0.02 |
| J5 | 180.07 | neg | L-Tyrosine | 4.66±0.02 | 4.35±0.01 | 4.63±0.08 |
| J6 | 205.03 | neg | Homocitric acid | 4.00±0.02 | 4.58±0.04 | 5.01±0.04 |
| J7 | 171.08 | neg | Glycylproline | 5.12±0.01 | 5.15±0.01 | 5.16±0.02 |
| J8 | 284.22 | neg | Myristoylglycine | 4.28±0.03 | 4.40±0.13 | 4.30±0.08 |
| J9 | 191.02 | neg | Citric acid | 5.77±0.01 | 5.86±0.02 | 6.00±0.02 |
| J10 | 331.07 | neg | Gamma-Glutamylphenylalanine | 4.33±0.03 | 3.97±0.05 | 3.82±0.08 |
| J11 | 259.11 | neg | Tyrosyl-Proline | 4.18±0.03 | 4.16±0.05 | 4.19±0.06 |
| J12 | 639.16 | neg | Rhamnetin 3-laminaribioside | 5.23±0.03 | 3.95±0.04 | 3.98±0.03 |
| J13 | 263.07 | neg | Gamma-Glutamyl-S-methylcysteine | 5.76±0.00 | 5.81±0.00 | 5.83±0.03 |
| J14 | 157.05 | neg | Succinylacetone | 2.46±0.07 | 3.62±0.02 | 3.85±0.06 |
| J15 | 169.01 | neg | Cis-2-Methylaconitate | 3.82±0.06 | 3.98±0.05 | 4.08±0.03 |
| J16 | 227.10 | neg | L-beta-aspartyl-L-leucine | 3.08±0.03 | 3.25±0.01 | 3.89±0.07 |
| J17 | 128.03 | neg | Pyrroline hydroxycarboxylic acid | 4.42±0.08 | 4.35±0.17 | 4.42±0.04 |
| J18 | 131.03 | neg | Glutaric acid | 4.53±0.02 | 4.47±0.02 | 4.50±0.01 |
| J19 | 288.12 | neg | Ophthalmic acid | 2.97±0.06 | 3.68±0.06 | 4.13±0.05 |
| J20 | 150.04 | neg | L-Serine | 3.48±0.06 | 3.65±0.05 | 3.79±0.03 |
| J21 | 142.05 | neg | Acetylhomoserine | 4.30±0.01 | 4.34±0.02 | 4.38±0.02 |
| J22 | 145.01 | neg | Oxoglutaric acid | 3.16±0.03 | 3.67±0.03 | 3.91±0.03 |
| J23 | 188.06 | neg | N-Acetyl-L-glutamic acid | 3.29±0.03 | 3.88±0.02 | 4.09±0.05 |
| J24 | 263.01 | neg | Mevalonic acid-5P | 2.01±0.05 | 3.96±0.02 | 3.83±0.01 |
| J25 | 203.02 | neg | Daucic acid | 4.94±0.01 | 4.94±0.01 | 4.94±0.02 |
| J26 | 129.02 | neg | Gamma-delta-Dioxovaleric acid | 4.62±0.01 | 5.19±0.01 | 5.22±0.01 |
| J27 | 205.03 | neg | Oxoadipic acid | 5.00±0.03 | 4.84±0.03 | 4.83±0.02 |
| J28 | 171.03 | neg | 3-Dehydroquinate | 4.16±0.02 | 4.16±0.01 | 4.12±0.05 |
| J29 | 145.06 | neg | L-Glutamine | 4.40±0.12 | 4.66±0.05 | 4.86±0.05 |
| J30 | 127.05 | neg | Ureidoisobutyric acid | 3.79±0.02 | 4.17±0.01 | 4.49±0.04 |
| J31 | 275.12 | neg | Saccharopine | 4.16±0.00 | 4.26±0.06 | 5.24±0.09 |
| J32 | 173.10 | neg | L-Arginine | 3.69±0.02 | 4.15±0.04 | 4.42±0.07 |
| J33 | 154.06 | neg | 2-Oxoarginine | 4.25±0.09 | 4.39±0.03 | 4.45±0.08 |
| J34 | 134.05 | neg | Beta-Alanine | 4.08±0.01 | 2.15±0.06 | 3.23±0.12 |
| J35 | 143.00 | neg | 4-Hydroxy-2-oxoglutaric acid | 3.90±0.04 | 3.80±0.10 | 3.90±0.02 |
| J36 | 175.11 | pos | N2-Acetyl-L-ornithine | 5.65±0.03 | 5.46±0.02 | 5.44±0.04 |
| J37 | 102.06 | pos | L-Homoserine | 4.78±0.01 | 5.11±0.03 | 5.40±0.02 |
| J38 | 226.18 | pos | N-Undecanoylglycine | 5.83±0.04 | 5.95±0.12 | 5.90±0.05 |
| J39 | 189.12 | pos | Nepsilon-Acetyl-L-lysine | 4.55±0.05 | 4.53±0.05 | 4.70±0.05 |
| J40 | 208.10 | pos | N-Acetyl-L-phenylalanine | 3.97±0.01 | 4.09±0.06 | 4.14±0.05 |
| J41 | 108.08 | pos | L-2-Amino-3-methylenehexanoic acid | 4.23±0.01 | 4.12±0.02 | 4.11±0.01 |
| J42 | 101.07 | pos | L-2,4-diaminobutyric acid | 3.56±0.00 | 3.94±0.04 | 4.30±0.01 |
| J43 | 189.13 | pos | L-NMMA | 4.29±0.02 | 5.20±0.03 | 5.15±0.05 |
| J44 | 118.09 | pos | Betaine | 5.34±0.01 | 5.39±0.03 | 5.52±0.01 |
| J45 | 134.08 | pos | L-2-Amino-5-hydroxypentanoic acid | 4.54±0.01 | 4.42±0.01 | 4.59±0.01 |
| J46 | 217.12 | pos | Hydroxyvalerylglycine | 3.31±0.04 | 4.14±0.07 | 4.50±0.06 |
| J47 | 259.11 | pos | Tyrosyl-Hydroxyproline | 5.48±0.02 | 5.46±0.07 | 5.40±0.05 |
| J48 | 293.12 | pos | Glutamyltyrosine | 3.92±0.08 | 3.90±0.07 | 4.35±0.05 |
| J49 | 269.14 | pos | 3-Hydroxydodecanedioic acid | 4.12±0.02 | 4.14±0.03 | 4.15±0.04 |
| J50 | 189.15 | pos | Prenyl isobutyrate | 4.10±0.05 | 4.09±0.04 | 4.09±0.05 |
| J51 | 376.27 | pos | Kinetensin 1-3 | 4.14±0.01 | 4.14±0.04 | 4.14±0.03 |
| J52 | 308.09 | pos | Glutathione | 4.93±0.01 | 6.05±0.01 | 6.42±0.04 |
| J53 | 170.08 | pos | 2-Keto-6-acetamidocaproate | 4.66±0.02 | 3.82±0.06 | 4.43±0.06 |
| J54 | 170.04 | pos | 1-(Malonylamino)cyclopropanecarboxylic acid | 4.49±0.01 | 4.44±0.03 | 4.38±0.04 |
| J55 | 176.09 | pos | N-Carboxyethyl-gamma-aminobutyric acid | 5.42±0.03 | 6.25±0.04 | 6.30±0.02 |
| J56 | 114.06 | pos | 5-Aminolevulinic acid | 4.17±0.02 | 4.34±0.02 | 4.59±0.01 |
| J57 | 117.09 | pos | Ethyl 2-methylpropanoate | 3.44±0.02 | 2.55±0.05 | 2.37±0.12 |
| J58 | 124.11 | pos | DL-2-Aminooctanoic acid | 3.73±0.19 | 3.89±0.27 | 3.80±0.18 |
| J59 | 502.28 | pos | Dynorphin A 1-8 | 2.32±0.01 | 3.10±0.09 | 4.26±0.05 |
| J60 | 215.14 | pos | Threoninyl-Isoleucine | 3.68±0.04 | 3.64±0.05 | 4.53±0.10 |
| J61 | 210.08 | pos | Hydroxyphenylacetylglycine | 2.55±0.14 | 3.93±0.03 | 4.27±0.05 |
| J62 | 132.10 | pos | L-Alloisoleucine | 4.82±0.04 | 4.60±0.05 | 5.40±0.07 |
| J63 | 145.05 | pos | 3-Hydroxyadipic acid | 6.18±0.02 | 6.21±0.01 | 6.17±0.03 |
| J64 | 251.07 | pos | Gamma-Glu-Cys | 3.00±0.12 | 4.67±0.01 | 4.91±0.05 |
| J65 | 130.09 | pos | D-Pipecolic acid | 4.69±0.01 | 5.42±0.04 | 5.48±0.02 |
| J66 | 116.07 | pos | L-Proline | 6.10±0.02 | 6.05±0.01 | 6.10±0.02 |
| J67 | 284.29 | pos | Octadecanamide | 6.01±0.03 | 6.12±0.04 | 6.08±0.05 |
| J68 | 148.06 | pos | L-Glutamate | 4.73±0.05 | 5.02±0.05 | 5.26±0.05 |
| K1 | 324.29 | pos | Linoleoyl Ethanolamide | 5.31±0.03 | 5.28±0.07 | 5.18±0.09 |
| K2 | 318.30 | pos | Phytosphingosine | 5.74±0.01 | 5.93±0.02 | 6.01±0.04 |
| K3 | 272.26 | pos | C16 Sphingosine | 3.33±0.10 | 4.12±0.03 | 4.60±0.03 |
| K4 | 116.14 | pos | Isobutylpropylamine | 6.80±0.00 | 6.82±0.01 | 6.80±0.02 |
| K5 | 184.07 | pos | Phosphocholine | 4.41±0.03 | 3.15±0.11 | 3.16±0.02 |
| K6 | 302.30 | pos | Sphinganine | 5.23±0.06 | 5.14±0.04 | 5.17±0.03 |
| K7 | 120.08 | pos | 2-Hydroxyphenethylamine | 3.12±0.02 | 3.48±0.05 | 3.74±0.09 |
| K8 | 180.17 | pos | MEMANTINE | 4.27±0.04 | 4.40±0.12 | 4.37±0.06 |
| K9 | 131.13 | pos | Agmatine | 3.82±0.01 | 3.80±0.00 | 3.75±0.03 |
| K10 | 150.11 | pos | Trolamine | 5.49±0.00 | 5.50±0.03 | 5.47±0.03 |
| L1 | 165.04 | neg | 4-Deoxyerythronic acid | 5.85±0.02 | 5.80±0.00 | 5.82±0.01 |
| L2 | 369.14 | neg | Amylose | 5.16±0.03 | 5.42±0.01 | 5.34±0.03 |
| L3 | 195.05 | neg | Gluconic acid | 5.65±0.01 | 5.41±0.01 | 5.54±0.02 |
| L4 | 325.09 | neg | Trans-o-Coumaric acid 2-glucoside | 4.30±0.00 | 4.35±0.03 | 4.33±0.00 |
| L5 | 429.18 | neg | Phenethyl rutinoside | 5.10±0.01 | 5.16±0.03 | 5.15±0.02 |
| L6 | 365.15 | neg | Octanoylglucuronide | 4.82±0.02 | 4.59±0.05 | 4.16±0.09 |
| L7 | 173.04 | neg | Shikimic acid | 4.37±0.01 | 4.43±0.04 | 4.46±0.02 |
| L8 | 357.12 | neg | Moringyne | 4.08±0.04 | 4.04±0.02 | 3.90±0.05 |
| L9 | 427.18 | neg | 1,2,10-Trihydroxydihydro-trans-linalyl oxide 7-O-beta-D-glucopyranoside | 5.83±0.01 | 5.85±0.01 | 5.82±0.04 |
| L10 | 160.06 | neg | Glucosamine | 3.83±0.01 | 3.87±0.00 | 3.88±0.01 |
| L11 | 311.01 | neg | D-Sedoheptulose 7-phosphate | 4.56±0.01 | 4.61±0.03 | 4.89±0.04 |
| L12 | 533.17 | neg | Fucosyllactose | 5.22±0.11 | 4.67±0.03 | 5.29±0.05 |
| L13 | 505.18 | neg | (1R,2S,3R)-2-Acetyl-4(5)-(1,2,3,4-tetrahydroxybutyl)imidazole | 5.19±0.10 | 4.41±0.17 | 4.40±0.10 |
| L14 | 223.05 | neg | Gluconolactone | 4.41±0.19 | 4.54±0.06 | 4.62±0.03 |
| L15 | 151.06 | neg | Ribitol | 6.59±0.03 | 6.73±0.02 | 6.69±0.01 |
| L16 | 181.07 | neg | Dulcitol | 4.45±0.01 | 4.77±0.01 | 4.85±0.01 |
| L17 | 149.04 | neg | D-Ribose | 4.59±0.03 | 4.27±0.02 | 4.20±0.03 |
| L18 | 258.04 | neg | Glucosamine 6-phosphate | 3.71±0.26 | 4.67±0.09 | 4.91±0.08 |
| L19 | 214.05 | neg | Fructosamine | 3.88±0.02 | 5.41±0.02 | 5.63±0.03 |
| L20 | 193.03 | neg | Ribonolactone | 5.16±0.02 | 5.10±0.01 | 5.12±0.01 |
| L21 | 245.04 | neg | L-Galacto-2-heptulose | 5.23±0.02 | 5.67±0.03 | 5.65±0.02 |
| L22 | 245.03 | neg | L-Kynurenine | 4.53±0.02 | 4.47±0.02 | 4.61±0.05 |
| L23 | 191.06 | neg | D-altro-D-manno-Heptose | 7.57±0.02 | 7.49±0.00 | 7.51±0.01 |
| L24 | 221.03 | neg | Malonic semialdehyde | 4.58±0.03 | 4.56±0.02 | 4.62±0.01 |
| L25 | 199.04 | neg | L-Fucose | 4.60±0.04 | 4.58±0.02 | 4.61±0.03 |
| L26 | 313.11 | neg | 1-Deoxy-D-xylulose | 4.61±0.03 | 4.43±0.03 | 4.46±0.02 |
| L27 | 149.04 | neg | D-Apiose | 3.94±0.01 | 3.95±0.02 | 3.95±0.02 |
| L28 | 121.10 | pos | 3,5,5-Trimethyl-2-cyclohexen-1-one | 5.31±0.02 | 5.35±0.01 | 5.32±0.01 |
| L29 | 244.08 | pos | N-Acetylmannosamine | 5.41±0.01 | 5.22±0.02 | 5.23±0.01 |
| L30 | 527.16 | pos | 1-Kestose | 5.47±0.03 | 4.17±0.05 | 3.97±0.12 |
| L31 | 133.06 | pos | 4-Ipomeanol | 3.86±0.04 | 3.87±0.02 | 3.87±0.02 |
| L32 | 109.03 | pos | Quinone | 5.21±0.02 | 4.93±0.01 | 4.88±0.03 |
| L33 | 175.06 | pos | L-Arabitol | 6.88±0.01 | 7.03±0.02 | 6.93±0.03 |
| L34 | 226.22 | pos | (E)-6,10-Dimethyl-9-methylene-5-undecen-2-one | 5.19±0.03 | 5.24±0.01 | 5.23±0.05 |
| L35 | 70.07 | pos | 4-Aminobutyraldehyde | 4.82±0.02 | 4.71±0.01 | 4.77±0.03 |
| L36 | 184.17 | pos | 2,6,6-Trimethyl-1-cyclohexen-1-acetaldehyde | 5.17±0.03 | 5.23±0.02 | 5.22±0.04 |
| L37 | 144.10 | pos | Xi-4-Hydroxy-4-methyl-2-cyclohexen-1-one | 2.65±0.02 | 3.74±0.05 | 3.89±0.02 |
| L38 | 139.11 | pos | 2-Hydroxy-2,6,6-trimethylcyclohexanone | 6.28±0.02 | 6.29±0.02 | 6.28±0.01 |
| L39 | 325.14 | pos | DHAP(10:0) | 4.31±0.04 | 4.27±0.04 | 4.32±0.04 |
| L40 | 163.04 | pos | 5-Methylthioribose | 3.53±0.03 | 3.53±0.09 | 3.54±0.09 |
| L41 | 184.17 | pos | 2-Hexylidenecyclopentanone | 5.10±0.02 | 5.17±0.00 | 5.16±0.05 |
| L42 | 189.09 | pos | Dhelwangin | 4.25±0.00 | 4.27±0.01 | 4.24±0.02 |
| L43 | 127.11 | pos | Methylheptenone | 5.16±0.01 | 5.23±0.01 | 5.20±0.01 |
| L44 | 332.13 | pos | Vanilloside | 4.85±0.03 | 4.79±0.03 | 4.74±0.04 |
| L45 | 399.14 | pos | 5-Hydroxykynurenamine | 4.25±0.08 | 5.69±0.04 | 5.91±0.01 |
| L46 | 99.04 | pos | Deoxyribose | 5.80±0.01 | 5.83±0.01 | 5.79±0.02 |
| L47 | 149.02 | pos | Benzoquinoneacetic acid | 5.33±0.01 | 5.34±0.01 | 5.31±0.01 |
| L48 | 240.20 | pos | Isokobusone | 4.16±0.03 | 4.14±0.07 | 4.07±0.08 |
| L49 | 107.09 | pos | 2,3-Dimethyl-2-cyclohexen-1-one | 4.26±0.04 | 4.29±0.05 | 4.28±0.04 |
| L50 | 432.24 | pos | 4-(Methylnitrosamino)-1-(3-pyridyl)-1-butanone | 5.88±0.02 | 5.90±0.01 | 5.89±0.05 |
| L51 | 271.19 | pos | Alpha-Butyl-omega-hydroxypoly(oxyethylene) poly(oxypropylene) | 4.02±0.02 | 4.03±0.00 | 4.02±0.01 |
| L52 | 334.22 | pos | Cis-4-Hydroxycyclohexylacetic acid | 3.86±0.19 | 3.94±0.02 | 3.88±0.11 |
| L53 | 180.09 | pos | Beta-D-Glucosamine | 4.69±0.04 | 4.61±0.02 | 4.60±0.06 |
| L54 | 207.14 | pos | Eremopetasinorone A | 5.13±0.04 | 5.11±0.05 | 5.13±0.04 |
| L55 | 163.06 | pos | D-Tagatose | 4.56±0.04 | 4.50±0.06 | 4.50±0.07 |
| L56 | 458.76 | pos | Tylosin | 2.90±0.49 | 3.79±0.07 | 4.59±0.03 |
| L57 | 487.21 | pos | Linalool oxide D 3-[apiosyl-(1->6)-glucoside] | 5.28±0.03 | 5.25±0.07 | 5.24±0.06 |
| L58 | 123.04 | pos | 4-Hydroxybenzaldehyde | 4.52±0.03 | 4.40±0.02 | 4.73±0.04 |
| L59 | 91.04 | pos | Glyceraldehyde | 4.21±0.03 | 4.23±0.02 | 4.20±0.02 |
| L60 | 291.10 | pos | Neohesperidose | 5.60±0.02 | 5.41±0.02 | 5.21±0.05 |
| L61 | 73.07 | pos | Butanone | 4.76±0.01 | 4.79±0.01 | 4.74±0.02 |
| L62 | 365.10 | pos | Sucrose | 6.17±0.03 | 5.62±0.05 | 5.85±0.07 |
| L63 | 220.12 | pos | Pantothenic Acid | 5.53±0.02 | 5.43±0.02 | 5.54±0.04 |
| L64 | 300.29 | pos | 3-ketosphinganine | 4.08±0.05 | 4.64±0.03 | 4.96±0.07 |
| M1 | 447.15 | neg | (Z)-Narceine imide | 4.61±0.01 | 4.64±0.04 | 4.59±0.05 |
| M2 | 135.04 | neg | 3-(5-Methyl-2-furyl)prop-2-enal | 4.35±0.01 | 4.36±0.04 | 4.32±0.03 |
| M3 | 203.08 | neg | (+/-)-Tryptophan | 4.10±0.03 | 3.42±0.03 | 4.22±0.12 |
| M4 | 174.08 | neg | 2-Pyrrolidineacetic acid | 2.99±0.04 | 3.33±0.02 | 4.03±0.02 |
| M5 | 147.03 | neg | D-Xylono-1,5-lactone | 4.05±0.02 | 5.04±0.01 | 5.09±0.02 |
| M6 | 175.02 | neg | L-Ascorbic acid | 6.03±0.08 | 6.04±0.12 | 5.91±0.06 |
| M7 | 173.01 | neg | Dehydroascorbic acid | 5.33±0.02 | 5.21±0.03 | 5.09±0.03 |
| M8 | 167.02 | neg | Uric acid | 3.81±0.02 | 3.83±0.01 | 3.79±0.01 |
| M9 | 383.11 | neg | S-Adenosylhomocysteine | 3.33±0.03 | 3.94±0.03 | 4.42±0.08 |
| M10 | 151.03 | neg | Xanthine | 2.83±0.05 | 4.21±0.04 | 4.55±0.06 |
| M11 | 134.05 | neg | Adenine | 3.78±0.02 | 4.05±0.02 | 4.50±0.05 |
| M12 | 112.08 | pos | N-Acetyl-2,3-dihydro-1H-pyrrole | 5.22±0.03 | 5.33±0.03 | 5.37±0.02 |
| M13 | 153.09 | pos | 1,4-Ipomeadiol | 3.89±0.10 | 3.97±0.19 | 3.90±0.11 |
| M14 | 188.07 | pos | 3-Pyridinebutanoic acid | 4.71±0.02 | 4.59±0.05 | 4.73±0.03 |
| M15 | 139.05 | pos | 4-Imidazolone-5-propionic acid | 4.15±0.02 | 4.13±0.05 | 4.14±0.04 |
| M16 | 209.15 | pos | (5R,6S)-5,6-Epoxy-7-megastigmen-9-one | 4.99±0.02 | 4.93±0.05 | 4.82±0.06 |
| M17 | 215.02 | pos | D-Glucaro-1,4-lactone | 4.79±0.03 | 4.83±0.05 | 4.92±0.03 |
| M18 | 104.05 | pos | (+/-)-2-Methylthiazolidine | 3.7±0.04 | 3.66±0.06 | 4.43±0.07 |
| M19 | 137.05 | pos | Hypoxanthine | 3.44±0.04 | 4.49±0.05 | 4.89±0.05 |
| M20 | 113.03 | pos | Uracil | 4.09±0.04 | 3.86±0.02 | 4.65±0.06 |
| M21 | 198.19 | pos | Cis-Quinceoxepane | 5.20±0.03 | 5.26±0.02 | 5.25±0.05 |
| M22 | 152.06 | pos | Guanine | 4.85±0.04 | 5.16±0.05 | 5.35±0.04 |
| M23 | 191.08 | pos | 6-Hydroxy-1H-indole-3-acetamide | 4.10±0.01 | 3.92±0.03 | 3.96±0.05 |
| M24 | 239.16 | pos | Dihydrozeatin | 5.02±0.00 | 5.05±0.01 | 5.02±0.00 |
| M25 | 142.12 | pos | PELLETIERINE | 4.72±0.02 | 4.77±0.00 | 4.76±0.03 |
| M26 | 129.05 | pos | (+/-)-Furaneol | 4.69±0.02 | 4.66±0.03 | 4.55±0.06 |
| M27 | 123.06 | pos | Isonicotineamide | 4.80±0.04 | 4.86±0.04 | 5.49±0.07 |
| M28 | 157.09 | pos | 2,5-Dimethyl-4-ethoxy-3(2H)-furanone | 3.99±0.02 | 3.58±0.04 | 3.27±0.07 |
| M29 | 126.09 | pos | 6-Acetyl-1,2,3,4-tetrahydropyridine | 4.34±0.05 | 4.33±0.05 | 4.47±0.04 |
| M30 | 219.11 | pos | N-Acetylserotonin | 1.94±0.23 | 3.59±0.08 | 3.93±0.03 |
| M31 | 118.07 | pos | Indole | 4.94±0.02 | 4.94±0.02 | 4.90±0.01 |
| M32 | 115.08 | pos | Gamma-Caprolactone | 3.65±0.02 | 3.67±0.02 | 3.69±0.03 |
| M33 | 174.09 | pos | 5-Methoxytryptophol | 5.11±0.05 | 5.60±0.03 | 5.33±0.02 |
| M34 | 166.07 | pos | 7-Methylguanine | 3.40±0.05 | 4.25±0.05 | 4.56±0.04 |
| M35 | 123.06 | pos | Niacinamide | 4.84±0.01 | 3.74±0.06 | 4.36±0.09 |
| M36 | 124.04 | pos | Isonicotinic acid | 3.66±0.02 | 5.08±0.03 | 5.12±0.04 |
| M37 | 112.05 | pos | Cytosine | 4.53±0.04 | 4.85±0.05 | 4.67±0.07 |
| M38 | 150.08 | pos | 7-Methyladenine | 4.20±0.02 | 4.61±0.04 | 4.78±0.04 |
| M39 | 121.03 | pos | Ascladiol | 4.81±0.01 | 4.89±0.01 | 4.84±0.02 |
| M40 | 158.09 | pos | V-PYRRO/NO | 4.15±0.02 | 4.44±0.02 | 4.68±0.02 |
| M41 | 254.25 | pos | Ambronide | 5.82±0.01 | 5.90±0.02 | 5.87±0.06 |
| N1 | 515.32 | neg | Carpaine | 4.21±0.01 | 3.11±0.18 | 2.44±0.09 |
| N2 | 305.07 | neg | EPIGALLOCATECHIN | 4.15±0.04 | 4.20±0.03 | 4.18±0.01 |
| N3 | 165.05 | pos | 2-Hydroxycinnamic acid | 5.39±0.03 | 5.28±0.03 | 5.62±0.05 |
| N4 | 146.09 | pos | 4-Guanidinobutanoic acid | 4.50±0.03 | 4.49±0.04 | 4.53±0.05 |
| N5 | 725.23 | pos | 2-Feruloyl-1-sinapoylgentiobiose | 5.80±0.04 | 5.79±0.07 | 5.75±0.05 |
| N6 | 203.14 | pos | Alpha-Amylcinnamaldehyde | 4.12±0.02 | 4.13±0.03 | 4.12±0.02 |
| N7 | 460.27 | pos | 3-O-Acetylepisamarcandin | 6.05±0.06 | 6.10±0.04 | 6.11±0.02 |
| N8 | 446.25 | pos | Austalide L | 5.94±0.03 | 5.99±0.07 | 5.95±0.04 |
| N9 | 465.10 | pos | Quercetin 3-O-glucoside | 5.20±0.02 | 5.21±0.08 | 5.19±0.07 |
| N10 | 229.10 | pos | Ozagrel | 4.83±0.04 | 4.80±0.08 | 4.79±0.05 |
| N11 | 225.08 | pos | Sinapic acid | 4.97±0.03 | 4.92±0.06 | 4.89±0.06 |
| N12 | 207.07 | pos | CITROPTEN | 5.29±0.03 | 5.26±0.05 | 5.21±0.05 |
| N13 | 165.05 | pos | M-Coumaric acid | 4.89±0.03 | 4.80±0.05 | 4.72±0.06 |
| N14 | 511.34 | pos | 16beta-Hydroxystellatogenin | 5.17±0.01 | 5.15±0.08 | 5.14±0.06 |
| N15 | 200.05 | pos | P-CHLOROPHENYLALANINE | 6.65±0.03 | 6.65±0.02 | 6.59±0.01 |
| O1 | 229.10 | neg | Harmalan | 4.83±0.02 | 4.87±0.05 | 4.84±0.04 |
| O2 | 201.10 | pos | Harmalol | 5.35±0.02 | 5.35±0.07 | 5.34±0.04 |
| O3 | 130.16 | pos | 1-Octene | 6.95±0.00 | 6.98±0.01 | 6.96±0.01 |
| O4 | 95.09 | pos | 2-Methyl-1,3-cyclohexadiene | 3.72±0.02 | 3.70±0.02 | 3.68±0.03 |
| O5 | 353.14 | neg | (-)-Arctigenin | 4.63±0.04 | 5.11±0.05 | 5.21±0.05 |

Note：Benzenoids (F1-F26), Lipids and lipid-like molecules (G1-G143), Nucleosides, nucleotides, and analogues (H1-H7), Organic acids and derivatives (J1-J68), Organic nitrogen compounds (K1-K10), Organic oxygen compounds (L1-L64), Organoheterocyclic compounds (M1-M41), Phenylpropanoids and polyketides (N1-N15), Others (O1-O5).

**Supplementary Table 2** Analysis of differential metabolites between fermentation 0 h and 9 h

| Code | Metabolite | M/Z | Mode | VIP | Content | |
| --- | --- | --- | --- | --- | --- | --- |
|  |  |  |  |  | 0 h | 9 h |
| 1 | PC(14:0/0:0) | 468.31 | pos | 3.23 | 5.38±0.04 | 3.37±0.17 |
| 2 | Glycerophosphocholine | 258.11 | pos | 2.66 | 5.65±0.01 | 7±0.04 |
| 3 | 1-Kestose | 527.16 | pos | 2.60 | 5.47±0.03 | 4.17±0.05 |
| 4 | Nicotinamide adenine dinucleotide (NAD) | 664.12 | pos | 2.96 | 3.37±0.05 | 5.06±0 |
| 5 | Herbacetin 7-methyl ether 8-sophoroside | 641.17 | pos | 3.87 | 5.37±0.05 | 2.48±0.19 |
| 6 | N-Nitrosoguvacine | 157.06 | pos | 3.12 | 3.38±0.06 | 5.25±0.03 |
| 7 | 3-Hydroxytetradecanedioic acid | 319.15 | pos | 3.11 | 2.05±0.01 | 3.91±0.05 |
| 8 | ADP | 428.04 | pos | 2.78 | 3.16±0.12 | 4.65±0.01 |
| 9 | PC(16:1/0:0) | 494.32 | pos | 2.85 | 6.89±0.04 | 5.33±0.08 |
| 10 | PC(18:2/0:0) | 520.34 | pos | 2.67 | 6.6±0.05 | 5.22±0.09 |
| 11 | Sn-glycero-3-Phosphoethanolamine | 238.04 | pos | 3.99 | 1.93±0.38 | 5.03±0.04 |
| 12 | PC(18:3/0:0) | 518.32 | pos | 2.76 | 5.22±0.04 | 3.75±0.03 |
| 13 | 5-Hydroxykynurenamine | 399.14 | pos | 2.73 | 4.25±0.08 | 5.69±0.04 |
| 14 | MG(16:1(9Z)/0:0/0:0) | 329.27 | pos | 2.81 | 5.25±0.04 | 3.73±0.14 |
| 15 | Phosphocholine | 184.07 | pos | 2.56 | 4.41±0.03 | 3.15±0.11 |
| 16 | N-Acetylserotonin | 219.11 | pos | 2.91 | 1.94±0.23 | 3.59±0.08 |
| 17 | Hydroxyphenylacetylglycine | 210.08 | pos | 2.66 | 2.55±0.14 | 3.93±0.03 |
| 18 | Gamma-Glu-Cys | 251.07 | pos | 2.94 | 3.00±0.12 | 4.67±0.01 |
| 19 | Isonicotinic acid | 124.04 | pos | 2.71 | 3.66±0.02 | 5.08±0.03 |
| 20 | PC(12:0/0:0) | 440.28 | pos | 3.49 | 4.54±0.04 | 2.18±0.25 |
| 21 | Rhamnetin 3-laminaribioside | 639.16 | neg | 2.52 | 5.23±0.03 | 3.95±0.04 |
| 22 | LysoPC(14:0/0:0) | 512.30 | neg | 2.83 | 4.76±0.03 | 3.16±0.04 |
| 23 | PE(18:3/0:0) | 474.26 | neg | 2.57 | 4.2±0.01 | 2.87±0.08 |
| 24 | 2-(3,5-dihydroxy-4-methoxyphenyl)-4H-chromen-4-one | 305.04 | neg | 3.14 | 1.67±0.13 | 3.64±0.05 |
| 25 | 6-(acetyloxy)-3,4,5-trihydroxyoxane-2-carboxylic acid | 257.03 | neg | 3.32 | 2.08±0.17 | 4.3±0.01 |
| 26 | Mevalonic acid-5P | 263.01 | neg | 3.12 | 2.01±0.05 | 3.96±0.02 |
| 27 | Fructosamine | 214.05 | neg | 2.77 | 3.88±0.02 | 5.41±0.02 |
| 28 | Cytidine | 242.08 | neg | 2.81 | 3.82±0.06 | 5.41±0.03 |
| 29 | Beta-Alanine | 134.05 | neg | 3.11 | 4.08±0.01 | 2.15±0.06 |
| 30 | Xanthine | 151.03 | neg | 2.63 | 2.83±0.05 | 4.21±0.04 |

**Supplementary Table 3** Analysis of differential metabolites between fermentation 9 h and 18 h

| Code | Metabolite | M/Z | Mode | VIP | Content | |
| --- | --- | --- | --- | --- | --- | --- |
|  |  |  |  |  | 9 h | 18 h |
| 1 | THTC | 133.03 | pos | 2.26 | 4.19±0.06 | 5.02±0.07 |
| 2 | Ile Gly Thr Ile | 403.25 | pos | 3.11 | 2.87±0.15 | 4.42±0.07 |
| 3 | 3-amino-2-naphthoic acid | 188.07 | pos | 2.25 | 4.35±0.05 | 5.16±0.08 |
| 4 | (+/-)-2-Methylthiazolidine | 104.05 | pos | 2.19 | 3.66±0.06 | 4.43±0.07 |
| 5 | 3-Hydroxytetradecanedioic acid | 319.15 | pos | 2.32 | 3.91±0.05 | 4.77±0.05 |
| 6 | Uracil | 113.03 | pos | 2.22 | 3.86±0.02 | 4.65±0.06 |
| 7 | N2-(D-1-Carboxyethyl)-L-lysine | 219.13 | pos | 2.48 | 3.11±0.09 | 4.1±0.08 |
| 8 | Tyr Leu | 295.17 | pos | 2.51 | 2.86±0.07 | 3.87±0.06 |
| 9 | Ile Pro Val | 328.22 | pos | 2.84 | 3.15±0.12 | 4.45±0.09 |
| 10 | Phe Ile | 279.17 | pos | 2.37 | 2.86±0.13 | 3.77±0.06 |
| 11 | Ile Leu | 245.19 | pos | 2.39 | 3.00±0.07 | 3.93±0.07 |
| 12 | Tylosin | 458.76 | pos | 2.23 | 3.79±0.07 | 4.59±0.03 |
| 13 | Dynorphin A 1-8 | 502.28 | pos | 2.68 | 3.10±0.09 | 4.26±0.05 |
| 14 | Succinoadenosine | 384.11 | pos | 2.14 | 4.31±0.01 | 5.06±0.12 |
| 15 | Threoninyl-Isoleucine | 215.14 | pos | 2.35 | 3.64±0.05 | 4.53±0.1 |
| 16 | Arg Leu | 288.20 | pos | 2.26 | 4.06±0.03 | 4.88±0.1 |
| 17 | L-Alloisoleucine | 132.10 | pos | 2.22 | 4.6±0.05 | 5.4±0.07 |
| 18 | Ile Arg | 288.20 | pos | 2.92 | 3.16±0.08 | 4.53±0.13 |
| 19 | 1-Palmitoylglycerophosphoinositol | 571.29 | neg | 2.10 | 6.09±0.03 | 5.49±0.11 |
| 20 | 1-(11Z-eicosenoyl)-glycero-3-phosphate | 509.29 | neg | 2.17 | 4.36±0.13 | 3.68±0.21 |
| 21 | PG(16:0/0:0)[U] | 483.27 | neg | 2.08 | 3.46±0.07 | 2.83±0.24 |
| 22 | Carpaine | 515.32 | neg | 2.20 | 3.11±0.18 | 2.44±0.09 |
| 23 | 3-Methyl-3-butenyl apiosyl-(1->6)-glucoside | 379.16 | neg | 2.45 | 3.58±0.03 | 2.78±0.12 |
| 24 | (+/-)-Tryptophan | 203.08 | neg | 2.45 | 3.42±0.03 | 4.22±0.12 |
| 25 | L-beta-aspartyl-L-leucine | 227.10 | neg | 2.19 | 3.25±0.01 | 3.89±0.07 |
| 26 | 2-(3,5-dihydroxy-4-methoxyphenyl)-4H-chromen-4-one | 305.04 | neg | 2.06 | 3.64±0.05 | 4.21±0.05 |
| 27 | 2-Pyrrolidineacetic acid | 174.08 | neg | 2.30 | 3.33±0.02 | 4.03±0.02 |
| 28 | Fucosyllactose | 533.17 | neg | 2.16 | 4.67±0.03 | 5.29±0.05 |
| 29 | Saccharopine | 275.12 | neg | 2.72 | 4.26±0.06 | 5.24±0.09 |
| 30 | Beta-Alanine | 134.05 | neg | 2.84 | 2.15±0.06 | 3.23±0.12 |

**Supplementary Table 4** Analysis of differential metabolites between fermentation 0 h and 18 h

| Code | Metabolite | M/Z | Mode | VIP | Content | |
| --- | --- | --- | --- | --- | --- | --- |
|  |  |  |  |  | 0 h | 18 h |
| 1 | PC(14:0/0:0) | 468.31 | pos | 2.39 | 5.38±0.04 | 3.75±0.08 |
| 2 | Glycerophosphocholine | 258.11 | pos | 2.33 | 5.65±0.01 | 7.19±0.01 |
| 3 | Ile Gly Thr Ile | 403.25 | pos | 2.52 | 2.59±0.28 | 4.42±0.07 |
| 4 | Nicotinamide adenine dinucleotide (NAD) | 664.12 | pos | 2.65 | 3.37±0.05 | 5.37±0.09 |
| 5 | Herbacetin 7-methyl ether 8-sophoroside | 641.17 | pos | 3.21 | 5.37±0.05 | 2.45±0.13 |
| 6 | 3-Hydroxytetradecanedioic acid | 319.15 | pos | 3.10 | 2.05±0.01 | 4.77±0.05 |
| 7 | ADP | 428.04 | pos | 2.43 | 3.16±0.12 | 4.84±0.11 |
| 8 | PC(16:1/0:0) | 494.32 | pos | 2.41 | 6.89±0.04 | 5.24±0.01 |
| 9 | PC(18:2/0:0) | 520.34 | pos | 2.45 | 6.6±0.05 | 4.88±0.08 |
| 10 | Sn-glycero-3-Phosphoethanolamine | 238.04 | pos | 3.39 | 1.93±0.38 | 5.22±0.02 |
| 11 | 5-Hydroxykynurenamine | 399.14 | pos | 2.42 | 4.25±0.08 | 5.91±0.01 |
| 12 | MG(16:1(9Z)/0:0/0:0) | 329.27 | pos | 2.49 | 5.25±0.04 | 3.49±0.14 |
| 13 | N-Acetylserotonin | 219.11 | pos | 2.64 | 1.94±0.23 | 3.93±0.03 |
| 14 | Tylosin | 458.76 | pos | 2.38 | 2.9±0.49 | 4.59±0.03 |
| 15 | Dynorphin A 1-8 | 502.28 | pos | 2.61 | 2.32±0.01 | 4.26±0.05 |
| 16 | Hydroxyphenylacetylglycine | 210.08 | pos | 2.45 | 2.55±0.14 | 4.27±0.05 |
| 17 | 4-Hydroxyisoleucine | 148.10 | pos | 2.34 | 2.79±0.03 | 4.35±0.05 |
| 18 | Gamma-Glu-Cys | 251.07 | pos | 2.59 | 3±0.12 | 4.91±0.05 |
| 19 | PC(12:0/0:0) | 440.28 | pos | 2.89 | 4.54±0.04 | 2.15±0.29 |
| 20 | Carpaine | 515.32 | neg | 2.61 | 4.21±0.01 | 2.44±0.09 |
| 21 | PG(16:0/0:0)[U] | 483.27 | neg | 2.55 | 4.54±0.03 | 2.83±0.24 |
| 22 | 3-Methyl-3-butenyl apiosyl-(1->6)-glucoside | 379.16 | neg | 2.40 | 4.28±0.04 | 2.78±0.12 |
| 23 | Succinylacetone | 157.05 | neg | 2.31 | 2.46±0.07 | 3.85±0.06 |
| 24 | 2-(3,5-dihydroxy-4-methoxyphenyl)-4H-chromen-4-one | 305.04 | neg | 3.12 | 1.67±0.13 | 4.21±0.05 |
| 25 | 6-(acetyloxy)-3,4,5-trihydroxyoxane-2-carboxylic acid | 257.03 | neg | 2.82 | 2.08±0.17 | 4.16±0.01 |
| 26 | Mevalonic acid-5P | 263.01 | neg | 2.65 | 2.01±0.05 | 3.83±0.01 |
| 27 | Fructosamine | 214.05 | neg | 2.60 | 3.88±0.02 | 5.63±0.03 |
| 28 | Cytidine | 242.08 | neg | 2.57 | 3.82±0.06 | 5.54±0.01 |
| 29 | Uridine monophosphate (UMP) | 323.03 | neg | 2.42 | 3.78±0.02 | 5.3±0.07 |
| 30 | Xanthine | 151.03 | neg | 2.57 | 2.83±0.05 | 4.55±0.06 |

**Supplementary** **Table 5** Content changes of lipids and lipid-like molecules during fermentation

| Code | Metabolite | | M/Z | | Mode | | Content | | | | | |  |
| --- | --- | --- | --- | --- | --- | --- | --- | --- | --- | --- | --- | --- | --- |
|  |  |  |  |  |  |  | 0 h | | 9 h | | 18 h | |  |
| A1 | | 6S,9R-Dihydroxy-4,7E-megastigmadien-3-one 9-[apiosyl-(1->6)-glucoside] | | 517.23 | | neg | | 4.84±0.02 | | 4.86±0.04 | | 4.88±0.02 | |
| A2 | | Corchoionol C 9-glucoside | | 431.19 | | neg | | 5.1±0.02 | | 5.12±0.02 | | 5.09±0.01 | |
| A3 | | (Z)-2-Methyl-2-butene-1,4-diol 4-O-beta-D-Glucopyranoside | | 309.12 | | neg | | 5.13±0.02 | | 5.12±0.03 | | 5.06±0.03 | |
| A4 | | 1-Hexanol arabinosylglucoside | | 441.20 | | neg | | 5.03±0.02 | | 5.11±0.02 | | 5.1±0.01 | |
| A5 | | LysoPC(16:1(9Z)/0:0) | | 538.32 | | neg | | 6.14±0.04 | | 4.98±0.07 | | 4.91±0.05 | |
| A6 | | LysoPC(16:0) | | 540.33 | | neg | | 6.21±0.02 | | 5.55±0.03 | | 5.21±0.11 | |
| A7 | | 2-Isopropylmalic acid | | 175.06 | | neg | | 4.47±0.01 | | 4.94±0.03 | | 5.14±0.04 | |
| A8 | | Isorhamnetin 3-glucoside | | 477.10 | | neg | | 6.47±0.03 | | 6.57±0.04 | | 6.56±0.02 | |
| A9 | | LysoPC(18:1(9Z)) | | 566.35 | | neg | | 5.95±0.02 | | 5.37±0.06 | | 4.99±0.1 | |
| A10 | | 1-(sn-Glycero-3-phospho)-1D-myo-inositol | | 333.06 | | neg | | 5.69±0.01 | | 6.47±0.01 | | 6.24±0.03 | |
| A11 | | 3-hydroxy-3-methyl-Glutaric acid | | 161.04 | | neg | | 4.59±0.03 | | 4.59±0.02 | | 4.63±0.02 | |
| A12 | | Pantoyllactone glucoside | | 337.11 | | neg | | 4.12±0.01 | | 4.14±0.02 | | 4.13±0.02 | |
| A13 | | 9,10,13-TriHOME | | 329.23 | | neg | | 4.35±0.02 | | 4.4±0.06 | | 4.42±0.06 | |
| A14 | | 4a-Carboxy-4b-methyl-5a-cholesta-8,24-dien-3b-ol | | 487.34 | | neg | | 4.76±0.02 | | 4.78±0.05 | | 4.86±0.04 | |
| A15 | | Cis-9,10-Epoxystearic acid | | 297.24 | | neg | | 4.41±0.01 | | 4.72±0.06 | | 4.64±0.08 | |
| A16 | | 3-Oxohexadecanoic acid | | 269.21 | | neg | | 4.22±0.01 | | 4.7±0.02 | | 4.79±0.06 | |
| A17 | | 3-O-cis-Coumaroylmaslinic acid | | 617.39 | | neg | | 4.58±0.03 | | 4.71±0.08 | | 4.83±0.09 | |
| A18 | | 1-Palmitoylglycerophosphoinositol | | 571.29 | | neg | | 5.33±0.02 | | 6.09±0.03 | | 5.49±0.11 | |
| A19 | | 1-(11Z-eicosenoyl)-glycero-3-phosphate | | 509.29 | | neg | | 3.95±0.02 | | 4.36±0.13 | | 3.68±0.21 | |
| A20 | | PA(18:1(9Z)/22:2(13Z,16Z)) | | 775.53 | | neg | | 4.46±0.03 | | 4.57±0.06 | | 4.5±0.06 | |
| A21 | | NORETHINDRONE ACETATE | | 339.20 | | neg | | 5.34±0.07 | | 5.39±0.06 | | 5.39±0.05 | |
| A22 | | Prostaglandin G2 2-glyceryl Ester | | 477.22 | | neg | | 4.01±0 | | 4.62±0.1 | | 4.2±0.08 | |
| A23 | | Ganoderiol I | | 523.34 | | neg | | 4.18±0.04 | | 4.21±0.05 | | 4.49±0.16 | |
| A24 | | LysoPA(0:0/18:2(9Z,12Z)) | | 433.24 | | neg | | 5.11±0.02 | | 5.14±0.03 | | 4.69±0.09 | |
| A25 | | 1-(9Z-hexadecenoyl)-glycero-3-phosphate | | 407.22 | | neg | | 5.1±0.03 | | 5.18±0.05 | | 4.72±0.07 | |
| A26 | | LysoPC(15:0) | | 480.31 | | neg | | 5.29±0.02 | | 4.59±0.03 | | 4.21±0.14 | |
| A27 | | PE(16:0/0:0) | | 452.28 | | neg | | 5.44±0.03 | | 5.19±0.01 | | 4.92±0.09 | |
| A28 | | 2-hydroxyhexadecanoic acid | | 271.23 | | neg | | 3.58±0.02 | | 4.62±0.07 | | 4.55±0.08 | |
| A29 | | Azukisapogenol | | 471.35 | | neg | | 4.6±0.03 | | 4.77±0.05 | | 5.03±0.12 | |
| A30 | | LysoPC(14:0/0:0) | | 512.30 | | neg | | 4.76±0.03 | | 3.16±0.04 | | 3.39±0.06 | |
| A31 | | 5,10-Pentadecadien-1-ol | | 269.21 | | neg | | 3.86±0.01 | | 4.12±0.1 | | 3.98±0.1 | |
| A32 | | Furanofukinin | | 293.18 | | neg | | 4.11±0 | | 4.14±0 | | 4.13±0 | |
| A33 | | Corchorifatty acid F | | 327.22 | | neg | | 4.04±0.04 | | 4.09±0.06 | | 4.08±0.04 | |
| A34 | | 3-Methyl-3-butenyl apiosyl-(1->6)-glucoside | | 379.16 | | neg | | 4.28±0.04 | | 3.58±0.03 | | 2.78±0.12 | |
| A35 | | 4-Ethoxy-4-oxobutanoic acid | | 351.13 | | neg | | 4.62±0.01 | | 4.66±0.05 | | 4.66±0.01 | |
| A36 | | (R)-1-O-[b-D-Glucopyranosyl-(1->6)-b-D-glucopyranoside]-1,3-octanediol | | 451.22 | | neg | | 4.05±0.02 | | 4.07±0.06 | | 4.15±0.02 | |
| A37 | | Ethyl (S)-3-hydroxybutyrate glucoside | | 293.12 | | neg | | 4.53±0.02 | | 4.54±0.03 | | 4.53±0.02 | |
| A38 | | Adipic acid | | 145.05 | | neg | | 3.65±0.17 | | 4.67±0.05 | | 4.73±0.02 | |
| A39 | | Ilicifolinoside A | | 309.12 | | neg | | 4.18±0.02 | | 4.18±0.03 | | 4.18±0.01 | |
| A40 | | 1-O-alpha-D-Glucopyranosyl-D-mannitol | | 365.11 | | neg | | 5.25±0.01 | | 5.27±0 | | 5.29±0.02 | |
| A41 | | PC(16:0/0:0) | | 496.34 | | pos | | 6.93±0.03 | | 5.97±0.1 | | 5.56±0.11 | |
| A42 | | Germacrenone | | 256.23 | | pos | | 4.73±0.02 | | 4.79±0.02 | | 4.8±0.08 | |
| A43 | | Sn-glycero-3-Phosphoethanolamine | | 238.04 | | pos | | 1.93±0.38 | | 5.03±0.04 | | 5.22±0.02 | |
| A44 | | 8(R)-Hydroperoxylinoleic acid | | 335.22 | | pos | | 5.61±0.01 | | 5.66±0.01 | | 5.63±0.02 | |
| A45 | | Propyl 2,4-decadienoate | | 228.20 | | pos | | 6.24±0.01 | | 6.28±0.01 | | 6.29±0.05 | |
| A46 | | Tanacetin | | 247.13 | | pos | | 4.45±0.04 | | 4.42±0.09 | | 4.41±0.06 | |
| A47 | | Ethyl 2-furanacrylate | | 131.05 | | pos | | 5.49±0.06 | | 4.87±0.03 | | 5.12±0.06 | |
| A48 | | (2E,4E)-2,4-Hexadienoic acid | | 95.05 | | pos | | 5.13±0.02 | | 5.2±0.02 | | 5.14±0.03 | |
| A49 | | Maleamic acid | | 133.06 | | pos | | 6.02±0.02 | | 5.68±0.01 | | 5.65±0.03 | |
| A50 | | Glycerophosphocholine | | 258.11 | | pos | | 5.65±0.01 | | 7±0.04 | | 7.19±0.01 | |
| A51 | | 4,5-Dihydrovomifoliol | | 209.15 | | pos | | 4.48±0.02 | | 4.49±0.04 | | 4.48±0.04 | |
| A52 | | 1-(3-Methylbutanoyl)-6-apiosylglucose | | 419.15 | | pos | | 5.7±0.04 | | 5.66±0.06 | | 5.66±0.04 | |
| A53 | | Norecasantalic acid | | 181.12 | | pos | | 5.09±0.01 | | 5.06±0.06 | | 4.94±0.09 | |
| A54 | | Beta-Ionol | | 212.20 | | pos | | 5.21±0.03 | | 5.27±0.01 | | 5.26±0.05 | |
| A55 | | 3-Methylglutaric acid | | 129.05 | | pos | | 5.72±0 | | 5.75±0.01 | | 5.75±0.01 | |
| A56 | | DG(18:0/18:2(9Z,12Z)/0:0) | | 638.57 | | pos | | 6.01±0.02 | | 6.02±0.02 | | 6.03±0.02 | |
| A57 | | 9,10-DHOME | | 315.25 | | pos | | 7.41±0.02 | | 7.42±0.02 | | 7.42±0.02 | |
| A58 | | 5-Hydroxy-p-mentha-6,8-dien-2-one | | 131.09 | | pos | | 5.22±0.04 | | 5.22±0.04 | | 5.22±0.03 | |
| A59 | | Palmitic amide | | 256.26 | | pos | | 6.66±0.02 | | 6.74±0.02 | | 6.69±0.05 | |
| A60 | | 4-Isopropyl-3-cyclohexene-1-carboxylic acid | | 133.10 | | pos | | 4.39±0.01 | | 4.41±0.02 | | 4.41±0.04 | |
| A61 | | 10-Undecenyl acetate | | 177.16 | | pos | | 5.15±0.02 | | 5.14±0.05 | | 5.12±0.06 | |
| A62 | | Cynaroside A | | 467.19 | | pos | | 5.02±0.04 | | 4.99±0.03 | | 5±0.01 | |
| A63 | | Methyl 3-(methylthio)butanoate | | 335.07 | | pos | | 4.95±0.02 | | 5.72±0.02 | | 5.39±0.07 | |
| A64 | | 2-Pentenoic acid | | 83.05 | | pos | | 4.81±0.02 | | 4.88±0.01 | | 4.83±0.03 | |
| A65 | | Isopentyl beta-D-glucoside | | 251.15 | | pos | | 5.11±0.03 | | 5.06±0.05 | | 5.05±0.06 | |
| A66 | | Menthadienyl acetate | | 212.16 | | pos | | 3.7±0.03 | | 3.83±0.12 | | 3.9±0.29 | |
| A67 | | 3-Oxo-alpha-ionol 9-[apiosyl-(1->6)-glucoside] | | 525.23 | | pos | | 4.69±0.03 | | 4.66±0.13 | | 4.75±0.08 | |
| A68 | | (3b,9R)-5-Megastigmene-3,9-diol 9-[apiosyl-(1->6)-glucoside] | | 529.26 | | pos | | 4.93±0.04 | | 4.88±0.08 | | 4.9±0.07 | |
| A69 | | PC(12:0/0:0) | | 440.28 | | pos | | 4.54±0.04 | | 2.18±0.25 | | 2.15±0.29 | |
| A70 | | 2-DECENOL | | 330.34 | | pos | | 5.02±0.03 | | 4.95±0.01 | | 4.92±0.01 | |
| A71 | | DG(8:0/15:0/0:0) | | 465.35 | | pos | | 5.87±0.01 | | 5.93±0.02 | | 5.93±0.01 | |
| A72 | | (9R,10S,12Z)-9,10-Dihydroxy-8-oxo-12-octadecenoic acid | | 311.22 | | pos | | 5.3±0.01 | | 5.36±0.02 | | 5.36±0.02 | |
| A73 | | PC(16:0/16:1(9Z)) | | 732.55 | | pos | | 5.75±0.58 | | 6.16±0.04 | | 5.76±0.59 | |
| A74 | | 2-Hydroxypropyl 2-isopropyl-5-methylcyclohexyl carbonate | | 259.19 | | pos | | 5.84±0.01 | | 5.87±0 | | 5.87±0.01 | |
| A75 | | Araliacerebroside | | 732.56 | | pos | | 5.45±0.08 | | 5.48±0.07 | | 5.48±0.09 | |
| A76 | | (+/-)-1,4-Nonanediol diacetate | | 245.17 | | pos | | 5.55±0.06 | | 5.56±0.08 | | 5.59±0.05 | |
| A77 | | DG(18:1(9Z)/18:3(6Z,9Z,12Z)/0:0) | | 617.51 | | pos | | 5.14±0.05 | | 5.15±0.08 | | 5.13±0.07 | |
| A78 | | DG(18:3(6Z,9Z,12Z)/16:1(9Z)/0:0) | | 589.48 | | pos | | 5.06±0.03 | | 5.11±0.04 | | 5.01±0.01 | |
| A79 | | Azelaic acid | | 171.10 | | pos | | 4.41±0.02 | | 4.5±0.04 | | 4.39±0.01 | |
| A80 | | (S)-(-)-Perillyl alcohol | | 170.15 | | pos | | 5.04±0.04 | | 5.09±0.01 | | 5.08±0.04 | |
| A81 | | (R)-Carvotanacetone | | 170.15 | | pos | | 4.88±0.02 | | 4.96±0.01 | | 4.94±0.05 | |
| A82 | | (E)-11-Hexadecenoic acid | | 237.22 | | pos | | 5.28±0.01 | | 5.3±0.01 | | 5.32±0.02 | |
| A83 | | LysoPE(16:1(9Z)/0:0) | | 452.28 | | pos | | 5.72±0.04 | | 4.86±0.07 | | 4.79±0.01 | |
| A84 | | PC(14:0/0:0) | | 468.31 | | pos | | 5.38±0.04 | | 3.37±0.17 | | 3.75±0.08 | |
| A85 | | 8-Deoxy-11,13-dihydroxygrosheimin | | 281.14 | | pos | | 5.34±0.05 | | 5.38±0.06 | | 5.37±0.05 | |
| A86 | | 2,5-Heptadien-1-ol | | 95.09 | | pos | | 4.13±0.01 | | 4.15±0 | | 4.15±0.04 | |
| A87 | | 2-(3-Hydroxy-4-methylphenyl)-5-methyl-4-hexen-3-one | | 219.14 | | pos | | 4.13±0.02 | | 4.13±0.01 | | 4.09±0.04 | |
| A88 | | (S)-Batatic acid | | 197.08 | | pos | | 5.06±0.02 | | 5.05±0.06 | | 5.05±0.06 | |
| A89 | | (+)-Abscisic Acid | | 265.14 | | pos | | 4.81±0.03 | | 4.81±0.04 | | 4.77±0.04 | |
| A90 | | Glycerol 1-propanoate diacetate | | 197.08 | | pos | | 3.8±0.02 | | 3.79±0.04 | | 3.77±0.07 | |
| A91 | | 3-Hydroxytetradecanedioic acid | | 319.15 | | pos | | 2.05±0.01 | | 3.91±0.05 | | 4.77±0.05 | |
| A92 | | Kojibiose | | 325.11 | | pos | | 4.41±0.05 | | 5.11±0.05 | | 5±0.04 | |
| A93 | | Ethyl 3-hydroxydodecanoate | | 262.24 | | pos | | 4.13±0.1 | | 4.06±0.07 | | 4.18±0.03 | |
| A94 | | 1-Hexanol | | 246.24 | | pos | | 4.75±0.08 | | 4.62±0.07 | | 4.73±0.02 | |
| A95 | | 10,20-Dihydroxyeicosanoic acid | | 362.33 | | pos | | 4.97±0.17 | | 5.1±0.17 | | 5.04±0.01 | |
| A96 | | (-)-Carvomenthone | | 172.17 | | pos | | 4.75±0 | | 4.78±0 | | 4.74±0 | |
| A97 | | Polyoxyethylene 40 monostearate | | 346.33 | | pos | | 4.75±0.11 | | 4.69±0.06 | | 4.75±0.03 | |
| A98 | | (13R,14R)-8-Labdene-13,14,15-triol | | 342.30 | | pos | | 4.48±0.02 | | 5.23±0.01 | | 4.83±0.06 | |
| A99 | | Butyl dodecanoate | | 320.26 | | pos | | 4.65±0 | | 4.74±0.03 | | 4.74±0.06 | |
| A100 | | PC(18:3/0:0) | | 518.32 | | pos | | 5.22±0.04 | | 3.75±0.03 | | 3.74±0.06 | |
| A101 | | Decenedioic acid | | 201.11 | | pos | | 4.94±0.01 | | 4.95±0.01 | | 4.92±0.01 | |
| A102 | | Tridecanol | | 242.25 | | pos | | 4.46±0.02 | | 4.54±0.02 | | 4.51±0.07 | |
| A103 | | Polyporusterone A | | 479.33 | | pos | | 5.89±0.01 | | 5.93±0.01 | | 5.92±0.01 | |
| A104 | | (3E,5Z)-3,5-Octadien-1-ol | | 109.10 | | pos | | 4.41±0.04 | | 4.45±0 | | 4.45±0.03 | |
| A105 | | Serratol | | 323.29 | | pos | | 5.23±0.02 | | 5.27±0.03 | | 5.26±0.05 | |
| A106 | | MG(0:0/22:6(4Z,7Z,10Z,13Z,16Z,19Z)/0:0) | | 441.24 | | pos | | 5±0.11 | | 4.95±0.05 | | 5.16±0.16 | |
| A107 | | DG(18:0/18:3(6Z,9Z,12Z)/0:0) | | 619.53 | | pos | | 5.32±0.05 | | 5.29±0.02 | | 5.3±0.02 | |
| A108 | | Sebacic acid | | 203.13 | | pos | | 6.94±0.02 | | 6.95±0.02 | | 6.95±0.01 | |
| A109 | | DG(18:4(6Z,9Z,12Z,15Z)/18:2(9Z,12Z)/0:0) | | 613.48 | | pos | | 4.83±0.05 | | 4.92±0.08 | | 4.75±0 | |
| A110 | | Cucurbic acid | | 245.17 | | pos | | 4.8±0.07 | | 4.88±0.04 | | 4.84±0.03 | |
| A111 | | Trans-Dec-2-enoic acid | | 363.25 | | pos | | 5.35±0.01 | | 5.35±0.03 | | 5.35±0.02 | |
| A112 | | Humulol | | 240.23 | | pos | | 4.83±0.03 | | 4.9±0.01 | | 4.88±0.06 | |
| A113 | | 3,4-Dimethyl-5-pentyl-2-furanpropanoic acid | | 477.32 | | pos | | 5.68±0.01 | | 5.72±0.02 | | 5.7±0.01 | |
| A114 | | (3Z,6Z)-3,6-Nonadien-1-ol | | 123.12 | | pos | | 4.39±0.01 | | 4.46±0 | | 4.45±0.04 | |
| A115 | | 9,10-DiHOME | | 337.23 | | pos | | 5.79±0.01 | | 5.84±0.01 | | 5.84±0.01 | |
| A116 | | Yucalexin P21 | | 337.27 | | pos | | 5.65±0.03 | | 5.26±0.01 | | 5.2±0.02 | |
| A117 | | MG(18:2(9Z,12Z)/0:0/0:0) | | 355.28 | | pos | | 5.09±0.04 | | 4.35±0.05 | | 4.29±0.02 | |
| A118 | | MG(16:1(9Z)/0:0/0:0) | | 329.27 | | pos | | 5.25±0.04 | | 3.73±0.14 | | 3.49±0.14 | |
| A119 | | Nepetaside | | 329.16 | | pos | | 4.58±0.03 | | 4.49±0.02 | | 4.47±0.03 | |
| A120 | | PE(16:1(9Z)/P-18:1(9Z)) | | 722.50 | | pos | | 5.4±0.01 | | 5.42±0.02 | | 5.4±0.01 | |
| A121 | | PE(16:0/20:2(11Z,14Z)) | | 766.53 | | pos | | 5.49±0.01 | | 5.51±0.02 | | 5.48±0.01 | |
| A122 | | MG(0:0/18:3(6Z,9Z,12Z)/0:0) | | 353.27 | | pos | | 5.03±0.04 | | 3.85±0.09 | | 3.77±0.1 | |
| A123 | | Palmitic acid | | 320.26 | | pos | | 4.68±0.02 | | 4.77±0.05 | | 4.74±0.07 | |
| A124 | | 2(R)-hydroxydocosanoic acid | | 374.36 | | pos | | 4.44±0.05 | | 4.37±0.05 | | 4.39±0.04 | |
| A125 | | Tanacetol A | | 277.18 | | pos | | 3.97±0.01 | | 4.32±0.06 | | 4.46±0.04 | |
| A126 | | 10-Epijunenol | | 240.23 | | pos | | 4.45±0.03 | | 4.45±0.06 | | 4.41±0.05 | |
| A127 | | Melleolide | | 418.22 | | pos | | 5.68±0.04 | | 5.65±0.03 | | 5.63±0.1 | |
| A128 | | Ethyl 2-methyl-3,4-pentadienoate | | 105.07 | | pos | | 5.37±0 | | 5.36±0.03 | | 5.37±0.07 | |
| A129 | | 11-Hydroxy-9-tridecenoic acid | | 229.18 | | pos | | 4.35±0.02 | | 4.4±0.01 | | 4.43±0.07 | |
| A130 | | (E)-4,8-Dimethyl-1,3,7-nonatriene | | 151.15 | | pos | | 4.18±0.02 | | 4.21±0.01 | | 4.23±0.05 | |
| A131 | | 6-O-Oleuropeoylsucrose | | 547.18 | | pos | | 5.42±0.04 | | 5.4±0.06 | | 5.36±0.06 | |
| A132 | | 3-Hydroxy-beta-ionone | | 209.15 | | pos | | 3.91±0.01 | | 3.92±0.03 | | 4.02±0.02 | |
| A133 | | Xi-3-(4-Isopropylphenyl)-2-methylpropanal | | 191.14 | | pos | | 3.71±0.03 | | 3.87±0.05 | | 4.01±0.03 | |
| A134 | | Oleamide | | 282.28 | | pos | | 4.85±0.15 | | 5.15±0.12 | | 5.18±0.13 | |
| A135 | | Methylsuccinic acid | | 133.05 | | pos | | 4.49±0.03 | | 4.46±0.05 | | 4.48±0.05 | |
| A136 | | Cuminaldehyde | | 149.10 | | pos | | 4.24±0.03 | | 4.22±0.04 | | 4.23±0.03 | |
| A137 | | Prenyl glucoside | | 213.11 | | pos | | 3.87±0.04 | | 3.79±0.07 | | 3.68±0.06 | |
| A138 | | 8-Oxodiacetoxyscirpenol | | 398.18 | | pos | | 4.26±0.08 | | 4.25±0.06 | | 4.18±0.06 | |
| A139 | | Alpha-Crocetin glucosyl ester | | 535.19 | | pos | | 5.24±0.1 | | 4.49±0.05 | | 4.82±0.07 | |
| A140 | | Dehydrovomifoliol | | 223.13 | | pos | | 4.46±0.02 | | 4.52±0.04 | | 4.38±0.03 | |
| A141 | | Dodecanedioic acid | | 231.16 | | pos | | 4.13±0.07 | | 4.06±0.11 | | 4.14±0.08 | |
| A142 | | Dodecanoic acid | | 218.21 | | pos | | 4.5±0.08 | | 4.39±0.04 | | 4.5±0.03 | |
| A143 | | S-(2-Methylpropionyl)-dihydrolipoamide-E | | 319.15 | | pos | | 4.53±0.04 | | 4.48±0.08 | | 4.6±0.02 | |

**Table 6** Content changes of Amino acids, peptides, and analogues during fermentation

| Code | | Metabolite | | M/Z | | Mode | | Content | | | | | |
| --- | --- | --- | --- | --- | --- | --- | --- | --- | --- | --- | --- | --- | --- |
|  |  |  |  |  |  |  |  | 0 h | | 9 h | | 18 h | |
| B1 | N-Carboxyethyl-gamma-aminobutyric acid | | 176.09 | | pos | | 5.42±0.03 | | 6.25±0.04 | | 6.3±0.02 | |  |
| B2 | N-Undecanoylglycine | | 226.18 | | pos | | 5.83±0.04 | | 5.95±0.12 | | 5.9±0.05 | |  |
| B3 | N2-Acetyl-L-ornithine | | 175.11 | | pos | | 5.65±0.03 | | 5.46±0.02 | | 5.44±0.04 | |  |
| B4 | L-Homoserine | | 102.06 | | pos | | 4.78±0.01 | | 5.11±0.03 | | 5.4±0.02 | |  |
| B5 | N-Acetyl-L-phenylalanine | | 208.10 | | pos | | 3.97±0.01 | | 4.09±0.06 | | 4.14±0.05 | |  |
| B6 | 1-(Malonylamino)cyclopropanecarboxylic acid | | 170.04 | | pos | | 4.49±0.01 | | 4.44±0.03 | | 4.38±0.04 | |  |
| B7 | 5-Aminolevulinic acid | | 114.06 | | pos | | 4.17±0.02 | | 4.34±0.02 | | 4.59±0.01 | |  |
| B8 | Nepsilon-Acetyl-L-lysine | | 189.12 | | pos | | 4.55±0.05 | | 4.53±0.05 | | 4.70±0.05 | |  |
| B9 | L-2-Amino-3-methylenehexanoic acid | | 108.08 | | pos | | 4.23±0.01 | | 4.12±0.02 | | 4.11±0.01 | |  |
| B10 | L-2,4-diaminobutyric acid | | 101.07 | | pos | | 3.56±0.00 | | 3.94±0.04 | | 4.30±0.01 | |  |
| B11 | L-NMMA | | 189.13 | | pos | | 4.29±0.02 | | 5.2±0.03 | | 5.15±0.05 | |  |
| B12 | Betaine | | 118.09 | | pos | | 5.34±0.01 | | 5.39±0.03 | | 5.52±0.01 | |  |
| B13 | L-2-Amino-5-hydroxypentanoic acid | | 134.08 | | pos | | 4.54±0.01 | | 4.42±0.01 | | 4.59±0.01 | |  |
| B14 | Hydroxyvalerylglycine | | 217.12 | | pos | | 3.31±0.04 | | 4.14±0.07 | | 4.5±0.06 | |  |
| B15 | Tyrosyl-Hydroxyproline | | 259.11 | | pos | | 5.48±0.02 | | 5.46±0.07 | | 5.4±0.05 | |  |
| B16 | Glutamyltyrosine | | 293.12 | | pos | | 3.92±0.08 | | 3.9±0.07 | | 4.35±0.05 | |  |
| B17 | Kinetensin 1-3 | | 376.27 | | pos | | 4.14±0.01 | | 4.14±0.04 | | 4.14±0.03 | |  |
| B18 | DL-2-Aminooctanoic acid | | 124.11 | | pos | | 3.73±0.19 | | 3.89±0.27 | | 3.8±0.18 | |  |
| B19 | Dynorphin A 1-8 | | 502.28 | | pos | | 2.32±0.01 | | 3.10±0.09 | | 4.26±0.05 | |  |
| B20 | Threoninyl-Isoleucine | | 215.14 | | pos | | 3.68±0.04 | | 3.64±0.05 | | 4.53±0.10 | |  |
| B21 | Hydroxyphenylacetylglycine | | 210.08 | | pos | | 2.55±0.14 | | 3.93±0.03 | | 4.27±0.05 | |  |
| B22 | L-Alloisoleucine | | 132.10 | | pos | | 4.82±0.04 | | 4.60±0.05 | | 5.40±0.07 | |  |
| B23 | Gamma-Glu-Cys | | 251.07 | | pos | | 3.00±0.12 | | 4.67±0.01 | | 4.91±0.05 | |  |
| B24 | D-Pipecolic acid | | 130.09 | | pos | | 4.69±0.01 | | 5.42±0.04 | | 5.48±0.02 | |  |
| B25 | L-Proline | | 116.07 | | pos | | 6.10±0.02 | | 6.05±0.01 | | 6.10±0.02 | |  |
| B26 | Glutathione | | 308.09 | | pos | | 4.93±0.01 | | 6.05±0.01 | | 6.42±0.04 | |  |
| B27 | L-Glutamate | | 148.06 | | pos | | 4.73±0.05 | | 5.02±0.05 | | 5.26±0.05 | |  |
| B28 | Gamma-Glutamyl-S-methylcysteine | | 263.07 | | neg | | 5.76±0.00 | | 5.81±0.00 | | 5.83±0.03 | |  |
| B29 | L-Asparagine | | 131.04 | | neg | | 6.18±0.02 | | 5.77±0.04 | | 5.73±0.01 | |  |
| B30 | L-Tyrosine | | 180.07 | | neg | | 4.66±0.02 | | 4.35±0.01 | | 4.63±0.08 | |  |
| B31 | Glycylproline | | 171.08 | | neg | | 5.12±0.01 | | 5.15±0.01 | | 5.16±0.02 | |  |
| B32 | Gamma-Glutamylphenylalanine | | 331.07 | | neg | | 4.33±0.03 | | 3.97±0.05 | | 3.82±0.08 | |  |
| B33 | Tyrosyl-Proline | | 259.11 | | neg | | 4.18±0.03 | | 4.16±0.05 | | 4.19±0.06 | |  |
| B34 | Pyrroline hydroxycarboxylic acid | | 128.03 | | neg | | 4.42±0.08 | | 4.35±0.17 | | 4.42±0.04 | |  |
| B35 | Ophthalmic acid | | 288.12 | | neg | | 2.97±0.06 | | 3.68±0.06 | | 4.13±0.05 | |  |
| B36 | Saccharopine | | 275.12 | | neg | | 4.16±0.00 | | 4.26±0.06 | | 5.24±0.09 | |  |
| B37 | L-Arginine | | 173.10 | | neg | | 3.69±0.02 | | 4.15±0.04 | | 4.42±0.07 | |  |
| B38 | Beta-Alanine | | 134.05 | | neg | | 4.08±0.01 | | 2.15±0.06 | | 3.23±0.12 | |  |
| B39 | Pantetheine | | 277.12 | | neg | | 3.12±0.01 | | 3.32±0.07 | | 3.84±0.04 | |  |
| B40 | Myristoylglycine | | 284.22 | | neg | | 4.28±0.03 | | 4.40±0.13 | | 4.30±0.08 | |  |
| B41 | L-Serine | | 150.04 | | neg | | 3.48±0.06 | | 3.65±0.05 | | 3.79±0.03 | |  |
| B42 | Acetylhomoserine | | 142.05 | | neg | | 4.30±0.01 | | 4.34±0.02 | | 4.38±0.02 | |  |
| B43 | N-Acetyl-L-glutamic acid | | 188.06 | | neg | | 3.29±0.03 | | 3.88±0.02 | | 4.09±0.05 | |  |
| B44 | L-Glutamine | | 145.06 | | neg | | 4.4±0.12 | | 4.66±0.05 | | 4.86±0.05 | |  |

**Table 7** Content changes of Benzenoids during fermentation

| Code | Metabolite | M/Z | Mode | Content | | |
| --- | --- | --- | --- | --- | --- | --- |
|  |  |  |  | 0 h | 9 h | 18 h |
| C1 | Phthalic acid Mono-2-ethylhexyl Ester | 279.16 | pos | 5.31±0.02 | 5.35±0.03 | 5.36±0.01 |
| C2 | 2,5-Dimethylbenzaldehyde | 135.08 | pos | 4.96±0.05 | 4.98±0.05 | 4.95±0.05 |
| C3 | P-Tolualdehyde | 121.06 | pos | 4.88±0.01 | 4.84±0.02 | 4.83±0.07 |
| C4 | Fenamic acid | 231.11 | pos | 5.56±0.03 | 5.54±0.07 | 5.55±0.06 |
| C5 | Avenanthramide L | 308.09 | pos | 4.17±0.05 | 5.03±0.01 | 5.43±0.05 |
| C6 | 4-Hydroxy-5-phenyltetrahydro-1,3-oxazin-2-one | 176.07 | pos | 4.45±0.02 | 4.39±0.03 | 4.35±0.04 |
| C7 | Dihydrocapsaicin | 325.25 | pos | 4.59±0.37 | 4.54±0.34 | 4.38±0.47 |
| C8 | Alpha-Methylstyrene | 119.09 | pos | 5.66±0.04 | 5.69±0.06 | 5.67±0.05 |
| C9 | Benzylformate | 137.06 | pos | 3.71±0.02 | 3.74±0.04 | 3.72±0.05 |
| C10 | 4-Propylphenol | 137.10 | pos | 4.37±0.02 | 4.28±0.04 | 4.14±0.07 |
| C11 | P-Mentha-1,3,5,8-tetraene | 133.10 | pos | 4.66±0.03 | 4.57±0.04 | 4.41±0.08 |
| C12 | Sinapyl alcohol | 211.10 | pos | 4.54±0.01 | 4.53±0.07 | 4.54±0.05 |
| C13 | Butylparaben | 236.13 | pos | 4.12±0.02 | 4.13±0.08 | 4.15±0.03 |
| C14 | Diphenylamine | 170.10 | pos | 4.16±0.01 | 4.19±0.04 | 4.1±0.02 |
| C15 | 4-Ethylphenol | 123.08 | pos | 4.19±0.02 | 4.19±0.03 | 4.18±0.03 |
| C16 | Phenylpyruvic acid | 147.04 | pos | 4.97±0.03 | 4.76±0.06 | 4.59±0.07 |
| C17 | Phenylacetic acid | 119.05 | pos | 3.73±0.06 | 3.52±0.05 | 3.38±0.05 |
| C18 | 2-Naphthylamine | 144.08 | pos | 4.35±0.04 | 4.23±0.08 | 4.26±0.04 |
| C19 | Bk-DMBDB | 236.13 | pos | 3.54±0 | 4.39±0.06 | 4.43±0.04 |
| C20 | Benzaldehyde | 107.05 | pos | 4.5±0.06 | 3.96±0.03 | 4.19±0.05 |
| C21 | Phenylacetaldehyde | 121.06 | pos | 4.86±0.01 | 4.87±0.03 | 4.88±0.02 |
| C22 | Gentisic acid | 153.02 | neg | 3.68±0.02 | 3.79±0.01 | 3.76±0.01 |
| C23 | 2-Dodecylbenzenesulfonic acid | 325.18 | neg | 5.6±0.08 | 5.7±0.08 | 5.67±0.04 |
| C24 | Atrolactic acid | 165.05 | neg | 4.64±0.01 | 4.79±0.04 | 4.89±0.03 |
| C25 | Vanillin | 151.04 | neg | 3.51±0.03 | 3.55±0.03 | 3.7±0.03 |
| C26 | (4-Ethoxyphenyl)urea | 405.18 | neg | 4.26±0.04 | 4.31±0.03 | 4.29±0.02 |
